# Harnessing the Complete Repertoire of Conventional Dendritic Cell Functions for Cancer Immunotherapy

**DOI:** 10.3390/pharmaceutics12070663

**Published:** 2020-07-14

**Authors:** Lukas Amon, Lukas Hatscher, Lukas Heger, Diana Dudziak, Christian H. K. Lehmann

**Affiliations:** 1 Laboratory of Dendritic Cell Biology, Department of Dermatology, University Hospital Erlangen, Friedrich-Alexander University of Erlangen-Nürnberg, Hartmannstraße 14, D-91052 Erlangen, Germany; lukas.amon@uk-erlangen.de (L.A.); lukas.hatscher@fau.de (L.H.); lukas.heger@uk-erlangen.de (L.H.); 2 Deutsches Zentrum Immuntherapie (DZI), D-91054 Erlangen, Germany; 3 Comprehensive Cancer Center Erlangen-European Metropolitan Area of Nuremberg (CCC ER-EMN), D-91054 Erlangen, Germany; 4 Medical Immunology Campus Erlangen, D-91054 Erlangen, Germany

**Keywords:** dendritic cells, T cells, cancer therapy, checkpoint inhibition, antigen targeting, vaccination, pattern recognition receptors, tumor microenvironment

## Abstract

The onset of checkpoint inhibition revolutionized the treatment of cancer. However, studies from the last decade suggested that the sole enhancement of T cell functionality might not suffice to fight malignancies in all individuals. Dendritic cells (DCs) are not only part of the innate immune system, but also generals of adaptive immunity and they orchestrate the de novo induction of tolerogenic and immunogenic T cell responses. Thus, combinatorial approaches addressing DCs and T cells in parallel represent an attractive strategy to achieve higher response rates across patients. However, this requires profound knowledge about the dynamic interplay of DCs, T cells, other immune and tumor cells. Here, we summarize the DC subsets present in mice and men and highlight conserved and divergent characteristics between different subsets and species. Thereby, we supply a resource of the molecular players involved in key functional features of DCs ranging from their sentinel function, the translation of the sensed environment at the DC:T cell interface to the resulting specialized T cell effector modules, as well as the influence of the tumor microenvironment on the DC function. As of today, mostly monocyte derived dendritic cells (moDCs) are used in autologous cell therapies after tumor antigen loading. While showing encouraging results in a fraction of patients, the overall clinical response rate is still not optimal. By disentangling the general aspects of DC biology, we provide rationales for the design of next generation DC vaccines enabling to exploit and manipulate the described pathways for the purpose of cancer immunotherapy in vivo. Finally, we discuss how DC-based vaccines might synergize with checkpoint inhibition in the treatment of malignant diseases.

## 1. Introduction

Since their discovery by Ralph Steinman in 1973, dendritic cells (DCs) have emerged as central regulators of adaptive immune responses. DCs are superior in priming and activating naïve T cells in response to invading pathogens, but also maintain tolerance to self-antigens [1,2,3]. These functional capacities highlight the potency of DCs for therapeutic applications. Together with B cells, Macrophages, Langerhans cells (LCs), and inflammatory/monocyte-derived DCs (iDCs/moDCs), *bona fide* DCs are recognized as professional antigen presenting cells (APCs).

LCs are the only professional antigen presenting cell type present in the healthy epidermis. Over several decades, LCs have served as a paragon for DC biology since LCs migrate to skin draining lymph nodes following encounters with environmental cues within the epidermis to stimulate T cell responses [4]. These include the cross-priming of cytotoxic CD8^+^ T cells and cross-tolerance, the induction of T helper type 17 (Th17) responses and follicular T helper cells [4,5,6,7,8,9]. However, LCs originate from the yolk sac rendering them a *bona fide* macrophage population [4,10].

While absent under steady-state conditions, the in situ development of inflammatory DCs (iDCs)/monocyte derived DCs (moDCs) from monocytes at the site of inflammation has been described in mice and men [11]. Dependent on the utilized model, murine moDCs were attributed multiple functions during the induction of T cell immune responses including antigen presentation and subsequent polarization of T helper type 1 (Th1) cells, T helper type 2 (Th2) cells and cross-presentation to cytotoxic CD8^+^ T cells [12,13,14,15,16]. Besides the emergence of moDCs in situ, cell culture protocols for the differentiation of moDCs from human blood or murine bone marrow were developed very early, thereby overcoming the issue of low primary DC accessibility and their sensitivity [17,18]. Even though this renders moDCs as an interesting research tool, it always should be proven if findings generated utilizing moDCs are translatable to primary DCs [19,20]. Moreover, moDCs are often just simply referred to as DCs making it hard for the reader to distinguish moDCs and primary DCs at the first glance. Thus, we will highlight studies solely based on the use of moDCs in the course of the manuscript.

Primary DCs can be separated into conventional/classical DCs (cDCs), comprising cDC1 and cDC2, and plasmacytoid DCs (pDCs) [1,2,21,22,23,24,25]. While pDCs are prime-time producers of type I interferons upon viral infections, cDCs are the main antigen presenters in the DC family. In the past, researchers also used the terms myeloid and lymphoid DCs, but these terms have been replaced as their actual ontogeny has been unveiled in elegant studies. Therefore, we will only use the cDC1/cDC2 nomenclature throughout this review. In general, the cDC1 and cDC2 subsets share a similar functional core program including the sampling of the surrounding, antigen uptake, maturation, and migration to secondary lymphoid organs as well as the processing and presentation of peptides on major histocompatibility complex (MHC) molecules to naïve T cells [1,2,21,24,26,27]. Of note, the cDC1 and cDC2 subsets have unique functions in driving different T cell response modules and display discrete surface marker expression owing to distinct transcriptional programs (summarized in [1,2,21,25]).

In mice and men, cDCs are characterized by MHC II (in humans human leukocyte antigen HLA-DR), while markers prominent for other lineages are absent (mouse: Ly-6G, Siglec-F; human: CD20, CD56; mouse and human: CD3, CD19, NKp46) [1,28,29,30,31,32,33]. Additionally most cDCs of both species display CD11c expression, even though, human cDC1 are only intermediate for this integrin [30,31]. Additionally, CD26 may be employed as a general cDC marker in mice across tissues [30]. Furthermore, murine and human cDC1 share the expression of XCR1, CLEC9A, BTLA and NECL2, whereas murine cDC1 are positive for CD24, CD8α (lymphoid tissues/resident DCs), CD103 (non-lymphoid tissues/migratory DCs), or CD207 (e.g., skin, lymph node, lung) and human cDC1 show expression of CD141 (BDCA-3) [1,29,30,31,34,35,36,37,38,39,40,41,42]. Of note, a specialized cDC2 subset found in murine or human gut can co-express CD103 and CD11b or CD172a (SIRPα), respectively [42,43,44]. Thus, CD103 expression has only a limited use in the distinction of cDC1 and cDC2 in the gut. On the other hand, murine and human cDC2 express CD172a [29,30,31,42]. Additionally, human cDC2 express CD1c (BDCA-1), CD1b, CD1a (skin), and CLEC10A, while murine cDC2 are positive for CD11b [29,30,31,34,42,45]. Even though murine migratory DCs share many of the typical markers with lymphoid tissue resident cDCs, their transcriptomic signature strongly differs as highlighted by Miller et al. providing a resource summarizing the transcriptome of different DC subpopulations from various murine organs [46]. Further, a recent study by the group of Lambrecht reveals the emergence of an inflammatory conventional DC2 population in different models of infection displaying an overlapping phenotype to cDC1 and in vivo derived moDCs [47]. Even though both, inflammatory cDC2 and moDCs, share the expression of CD64 (FcγRI), FcεRI (MAR-1) and CD172a, cDC2 can be demarcated by CD26 expression [47]. Thorough separation of inflammatory cDC2 led to a lack of APC function by the remaining moDCs [47]. On the other hand, inflammatory cDC2 were able to present antigens to CD4^+^ and CD8^+^ T cells in parallel [47]. A comprehensive summary of the cDC surface marker profile is shown in Table 1.

Murine pDCs express Siglec-H, B220 (CD45R), and CD317 (BST2; PDCA-1), while human pDCs can be identified by CD303 (BDCA-2), CD304 (BDCA-4) and CD123 surface expression [30,31,34,48,49,50]. As the description and delineation of DC subpopulations by surface markers is not complete and since the onset of the single-cell era somewhat not state-of-the-art, transcription factor dependency and the development of the single subsets need to be included in the full description of DC subsets. Because discussing this information would be beyond of the scope of this review, we would like to recommend reading other recent summaries about subpopulation definitions, ontogeny, and transcription factor dependency [1,2,21,22,25,51,52].

Murine cDC1 were demonstrated to excel in their capability to cross-prime cytotoxic CD8^+^ T lymphocyte (CTL) responses [39,53,54,55,56]. Together with their ability to induce T helper type 1 (Th1) responses, cDC1 are key drivers of adaptive immune responses directed against intracellular pathogens and tumors [36,57,58,59,60]. Under non-inflammatory conditions, cDC1 are able to convert naïve T cells into regulatory T cells (T_reg_ cells) or induce T cell unresponsiveness (anergy) [40,61,62,63]. On the other hand, cDC2 are major regulators of T helper type 2 (Th2) and 17 (Th17) responses, thereby acting in the defense against extracellular pathogens [64,65,66,67,68,69,70,71]. Additionally, cDC2 are able to expand previously primed T_reg_ cells [63]. This functional division of labor is achieved by distinct cDC1 and cDC2 cytokine secretion patterns allowing for the polarization of the respective T cell subsets. This cytokine production is controlled by the upstream expression and action of a distinct transcriptional profile including transcription factors, components of the antigen processing machinery, and pattern recognition receptors (PRR) on cDC1 or cDC2, respectively [25,55,72,73]. In contrast to cDCs, the general capacity of pDCs to induce responses of naïve CD4^+^ and CD8^+^ T cells is currently under debate. In the past, multiple studies indicated that pDCs may initiate responses of naive T cells in mice and men [74,75,76,77]. However, a uniform stimulatory capacity of pDCs was not observed across all studies. For instance, it has been demonstrated that pDCs are either dispensable or unable to induce responses from the naïve T cell repertoire or only prime helper CD4^+^ T cells in an organ-dependent manner [56,78,79].

## 2. The Enigmatic Role of Plasmacytoid DCs in the DC Continuum

Recent insights provide new possible explanations for the controversially discussed ability of pDCs to induce T cell responses. In the human blood, Villani et al. recently described a new DC population termed AS DCs. These cells are exhibiting expression of AXL and SIGLEC-6 (thus AS DCs are also called AXL^+^SIGLEC-6^+^ DCs) together with typically used pDC markers, such as CD123 (IL3RA), CD303 (BDCA-2) and CD304 (BDCA-4; NRP1; CLEC4C) [33]. Upon closer analysis by transcriptional profiling, it became clear that AS DCs quite resembled the transcriptional signature of pDCs. Functional analyses however revealed that AS DCs displayed low proliferative potential, while efficiently stimulating T cells. Exclusion of AS DCs from the pDC pool strongly reduced the ability of T cell activation [33]. Moreover, See et al. identified a pre-cDC population in human blood resembling AS DCs. This pre-cDC population gave rise to cDC1 or cDC2 and was even able to stimulate T cells [32]. Taken together, these findings suggest, that human AS DCs are *bona fide* cDC progenitors (markers for mature cDCs are listed in Table 1) and might be previously mistaken as pDCs in earlier studies as both populations express characteristic pDC markers and molecules for targeted approaches including CD123, CD303 and CD304 [32,33]. Since many of these molecules have been exploited for cellular enrichment or characterization procedures, a potential contamination of human pDCs with AS DCs might explain previous controversial observations regarding the capacity of pDCs to induce immune responses from naïve T cells.

After the identification of pre-cDCs/AS DCs in humans, which shared many phenotypic characteristics with pDCs, the search for a similar cell type in mice began. There, Brown et al. discovered that human AS DCs closely resembled a murine Siglec-H^+^ pre-cDC progenitor, thereby validating the presence of a splenic cDC progenitor sharing phenotypic characteristics with pDCs in mice and men [80]. This murine Siglec-H expressing progenitor has already been described before. While an early study indicated that ablation of Siglec-H expressing cells did not only affect the pDC compartment, Schlitzer et al. directly identified a Siglec-H^+^ DC progenitor with decreasing frequency from bone marrow, to blood and spleen. This population finally lost Siglec-H expression upon further differentiation to cDC1 and cDC2 [50,81]. Additionally, a murine pendant of human AS DCs was recently identified by the Idoyaga group in spleen and lymph nodes and was termed transitional DCs (tDCs) [82]. Even though murine tDCs did not express AXL, they also exhibited characteristic features of both, pDCs and cDCs. This finding correlated with expression of a mixed set of transcription factors generic for either pDCs or cDCs including E2-2 or IRF8 and Zbtb46 [82]. However, the type I interferon (IFN-I) production ability of tDCs was low, while this subset efficiently activated T cells [82]. Additionally, other recent studies utilizing single cell-omic approaches provided evidence that also the T cell stimulatory capacity of murine pDCs may be attributed to a pre-cDC contamination within the pDC pool, which has previously not been recognized owing to the lack of resolution and the high resemblance of pDCs and pre-cDCs [83,84].

With respect to earlier studies examining pDCs, human AS DCs and murine tDCs might account for the T cell stimulatory capacity described for pDCs. For instance, antigen targeting to murine pDCs via PDCA-1 was protective in models of viral infection and cancer [74]. As murine tDCs also express PDCA-1, although to a lower extend, it is possible that antigens were additionally addressed to tDCs [82]. On the other hand, a pioneer study by Tel et al. utilizing antigen-loading of autologous human pDCs for the treatment of melanoma patients indicated that pDCs are capable to initiate antigen-specific T cell responses [76]. During this study, patient pDCs were isolated directly from blood via BDCA-4 (alias NRP1; CD304). Since human AS DCs were found capable of BDCA-4 expression, isolated pDC fractions may have contained AS DCs [33]. Thus, future studies will be required to clarify, if the T cell stimulatory capacity is an intrinsic feature of pDC biology or has to be attributed to pre-cDC contaminations within the pDC pool. This is eminent as a recent study demonstrated that following environmental stimuli (even after exclusion of AS DCs), a stable specialization of the human pDC compartment occurs [85]. Three pDC subsets could be identified, which included: (I) a type I IFN producing subset, (II) a population that was resembling DC morphology and exerting adaptive immune functions including activation and induction of proliferation of CD4^+^ T cells and (III) an intermediate population that was acquiring innate and adaptive functions [85]. Therefore, antigen-targeting studies to sorted pre-cDC and pDC populations utilizing subset specific receptors might clarify the role of pDCs in the induction of T cell responses. Further, murine models allowing for specific depletion might be needed to stratify, if tDCs are *bona fide* members of the cDC or pDCs lineage.

**Table 1:** Summary of prominent markers, pattern recognition receptors and co-regulatory molecules expressed by cDC1 and cDC2 in mice and men.

**Subpopulation**	**Species**	**Marker**	**Level of Detection**	**Literature**
**cDC1**	**Mouse**	CD8α (splenic, resident cDC1)	Protein; RNA	[80,86]
		CD1d	Protein	[86]
		CD207; Langerin	Protein; RNA	[35,37,80,82]
		CD24	Protein	[30,35,82]
		CD103^+^CD11b^-^ migratory cDC1	Protein	[35,42]
	**Shared**	XCR1	Protein; RNA	[30,33,42,82,86,87,88,89]
		CADM1; TSLC1; NECL-2; IGSF4; SynCAM1;	Protein; RNA	[30,31,33,42,82,86,87,88,89]
		CLEC9A; DNGR-1; CD370	Protein; RNA	[24,28,29,31,33,41,42,46,80,82,87,89,90,91]
		BTLA	Protein; RNA	[30,40,42,86,90]
		CD26	Protein	[29,30,90]
		Sirpα; CD172a negative	Protein	[28,30,92]
		CD11c (human cDC1int)	Protein	[28,30,31,42,80,86,88]
		MHC-II; HLA-DR	Protein	[28,29,30,42,86]
	**Human**	CD141; BDCA-3	Protein	[28,31,34,82,87,90,92]
		CD103^+^Sirpα^-^ migratory cDC1	Protein	[42]
**cDC2**	**Mouse**	CD4 (lymphoid tissue)	Protein; RNA	[80,93]
		DCIR2 (lymphoid tissue)	Protein; RNA	[55]
		CD103^+^CD11b^+^ gut migratory cDC2	Protein	[42]
	**Shared**	Sirpα; CD172a	Protein	[28,29,30,31,35,42,80,82,86,88,92]
		XCR1 negative	Protein	[30]
		CD11b	Protein; RNA	[28,30,42,80,88]
		CD11c	Protein; RNA	[28,30,31,42,80,86,88]
		MHC-II; HLA-DR	Protein	[28,29,30,42,86]
	**Human**	CD1c; BDCA-1	Protein; RNA	[28,31,34,80,90,91,92]
		CD1a (skin)	Protein	[28,94]
		CLEC10A	Protein; RNA	[33,45]
		CD163	Protein	[90,92]
		CD1d	Protein; RNA	[33,90]
		FcεRIα	Protein; RNA	[33,80,90]
		CD103^+^Sirpα^+^ gut migratory cDC2	Protein	[42]
**Subpopulation**	**Species**	**TLRs**	**Level of Detection**	**Literature**
**cDC1**	**Mouse**	TLR2	Protein	[86]
		TLR4; CD284	RNA	[80,89]
		TLR11	RNA	[24,46,80,89]
		TLR12	RNA	[24,46,80,86,88,95]
		TLR13	Protein; RNA	[24,46,80,88]
		CD180; RP105	Protein	[86]
	**Shared**	TLR3	Protein; RNA	[42,80,87,88,89,92,96,97]
		TLR9	Protein; RNA	[24,46,88,89,98]
	**Human**	TLR6	RNA	[89]
		TLR10; CD290	Protein	[90]
**cDC2**	**Mouse**	TLR9	Protein; RNA	[24,46,80,88,98]
		TLR13	Protein; RNA	[24,46,88]
	**Shared**	TLR1	Protein; RNA	[80,86,89]
		TLR2; CD282	Protein; RNA	[31,80,86,89,92]
		TLR4; CD284	RNA	[80,89]
		TLR5	RNA	[80,89,96,98]
		TLR6	Protein; RNA	[80,86,89]
		TLR7	Protein; RNA	[24,46,80,88,89,91]
		TLR8	Protein; RNA	[80,89,91,92]
		CD180; RP105	Protein	[86,90,92]
	**Human**	-	-	-
**Subpopulation**	**Species**	**CLRs**	**Level of Detection**	**Literature**
**cDC1**	**Mouse**	CD207; Langerin	Protein	[35,86]
		CLEC2D	Protein	[86]
		CLEC4A; DCIR; CD367	RNA	[42]
	**Shared**	DEC205; CD205; Ly75	Protein; RNA	[24,31,35,42,46,55,82,86,88,90]
		CLEC1A	RNA	[42]
		CLEC9A; DNGR-1; CD370	Protein; RNA	[24,28,29,31,33,41,42,46,82,87,89,91]
		CLEC12A; MICL; KLRL1; CLL1	Protein; RNA	[24,46,86,99]
	**Human**	CD206; MMR (skin)	Protein	[28]
		Dectin-1; CLEC7A; CLECSF12; CD369	Protein	[28]
**cDC2**	**Mouse**	Dectin-2; CLEC6A; CLEC4N; CLECSF10	Protein; RNA	[55,88]
		CLEC4a2; DCIR1; CLECSF6	RNA	[55,80,96]
		CLEC4a3; DCIR3	RNA	[55]
		CLEC4a4; DCIR2	Protein; RNA	[24,46,55,86,88]
		DCAR	RNA	[55]
	**Shared**	CLEC4A; DCIR; CD367 (subtype not defined)	Protein; RNA	[33,42,80,86,96]
		Dectin-1; CLEC7A; CLECSF12; CD369	Protein; RNA	[28,55,86]
		CLEC12A	Protein	[80,100]
		CLEC13A; CD302	RNA	[96]
		CD209 (human gut); CD209a (mouse); DC-SIGN	Protein; RNA	[24,42,46,55,80,89,91]
	**Human**	CD206; MMR (skin)	Protein	[28,42,90]
		CD207; Langerin	Protein; RNA	[42,91,94]
		CLEC5A (thymic cDC2)	RNA	[31]
		CLEC10A	Protein; RNA	[31,33,42,45,89]
		CLEC11A; CLECSF3 (CD5 low cDC2)	RNA	[91]
		CLEC17A (CD5 high cDC2)	RNA	[91]
**Subpopulation**	**Species**	**FcγRs**	**Level of Detection**	**Literature**
**cDC1**	**Mouse**	FcγRIII; CD16	Protein; RNA	[56,80,96]
		FcγRIV; CD16.2	Protein	[56]
	**Shared**	FcγRI; CD64	Protein; RNA	[30,42,56]
		FcγRIIB; CD32b	Protein; RNA	[56,80,86,101]
	**Human**	FcγRIIA; CD32	RNA	[102]
**cDC2**	**Mouse**	FcγRIV; CD16.2	Protein	[56]
		FcγRI; CD64 negative	Protein; RNA	[30,56,80]
	**Shared**	FcγRIIB; CD32b	Protein; RNA	[33,42,56,80,86,89,102]
		FcγRIII; CD16; human FcγRIIIA	Protein; RNA	[80,101,102]
	**Human**	FcγRI; CD64	Protein; RNA	[89,101]
		FcγRIIA; CD32	Protein; RNA	[28,31,96,101,102]
**Subpopulation**	**Species**	**Intracellular Sensors**	**Level of Detection**	**Literature**
**cDC1**	**Mouse**	NLRP3; NALP3; Cryopyrin	Protein	[88]
	**Shared**			
	**Human**	NLRC5	RNA	[42]
		Caspase-1 low	RNA	[92]
		AIM2	RNA	[92]
		PYCARD	RNA	[92]
**cDC2**	**Mouse**	NOD-1; NLRC1	Protein; RNA	[24,46,88]
		CARD9	Protein	[88]
		STING	Protein	[88]
		MDA5	Protein	[88]
		RIG-I	Protein	[88]
		NLRC4	RNA	[24,46]
	**Shared**	NLRP3; NALP3; Cryopyrin	Protein; RNA	[33,42,88,92]
		Caspase-1 high	Protein; RNA	[42,88,92]
	**Human**	Caspase-8	RNA	[92]
		NAIP	RNA	[92]
		NLRC4	RNA	[92]
		NLRP1	RNA	[92]
		PYCARD	RNA	[92]
**Subpopulation**	**Species**	**Co-Regulatory Molecules**	**Level of Detection**	**Literature**
**cDC1**	**Mouse**	-	-	-
	**Shared**	CD80	Protein	[30,42,55,82,87]
		CD86	Protein; RNA	[31,33,42,55,80,82,87,89,96]
		CD40	Protein; RNA	[30,42,55,89]
		PD-L1; CD274	Protein	[28,30,82,87,89]
	**Human**	IDO-1	Protein; RNA	[31,33,42,80,89,92]
		IDO-2	RNA	[42]
**cDC2**	**Mouse**	-	-	-
	**Shared**	CD80	Protein	[30,42,55,82,87]
		CD86	Protein; RNA	[30,31,33,42,55,82,87,96]
		CD40	Protein	[30,42,55,89]
		PD-L1; CD274	Protein	[30,80,82,87,89]
	**Human**	ICOS-L	RNA	[28]
**Subpopulation**	**Species**	**Other Interesting Molecules**	**Level of Detection**	**Literature**
**cDC1**	**Mouse**	CD36 (Scavenger receptor)	Protein	[86,88]
	**Shared**	CD135; FLT3	Protein; RNA	[28,80,91]
	**Human**	TIM-3	Protein	[90]
**cDC2**	**Mouse**	Clec12A (marker for cDC2B)	Protein	[80]
		Clec10A (marker for cDC2B)	Protein	[80]
		ESAM (marker for cDC2A or Notch2-dependent cDC2)	Protein	[66,68,80]
	**Shared**	CD135; FLT3	Protein; RNA	[28,80,91]
	**Human**	CD36 (marker for DC3; DC2 negative)	RNA	[31,33]
		CD163 (marker for DC3; DC2 negative)	Protein; RNA	[28,33]
		CD5 (marker for DC2; DC3 negative)	Protein; RNA	[90,91]
		CD200R (downregulating DC activity)	Protein	[90]

## 3. Environmental Cues Controlling the Stimulatory and Tolerogenic Capacity of DCs

DCs cannot only induce protective immune responses; they are also responsible for the perpetuation of tolerance to self-antigens [103,104,105,106]. Therefore, DCs are able to sense and react to a multitude of cues from the surroundings allowing them to differentiate, if a tolerogenic or protective immune response is required.

### 3.1. Dendritic Cells Function as Sensors for Invading Pathogens

DCs act as sentinels for pathogen entry across various tissues [21,29,30,31,42]. For this purpose, DCs are equipped with germline-encoded pattern recognition receptors (PRRs) allowing for the detection of conserved pathogen-associated molecular patterns (PAMPs) from viruses, bacteria, fungi, protozoa, and helminths [107,108,109]. Furthermore, many PRRs are able to recognize endogenous ligands, which are released from dying cells or following cellular stress. Such molecules are also known as danger associated molecular patterns (DAMPs) [110]. As induction of inflammation under sterile conditions is a prerequisite for the immune systems’ anti-cancer activity, DAMPs are key to allow for efficient induction of T cell immunity by DCs [110]. In general, ligand recognition by PRRs expressed on DCs can lead to DC activation and can induce simultaneous antigen uptake and processing [1,2,109]. Following antigen uptake in an inflammatory context, DCs upregulate co-stimulatory molecules (CD80, CD86) while processing the engulfed antigens and migrate to secondary lymphoid organs, a process driven by changes in their chemokine receptor expression profile (e.g., CCR7). There, they secrete specific cytokines and present the antigenic peptides as peptide: MHC complexes (pMHC) to T cells [1,2,21,109]. A summary of PRR expression on cDC subsets is shown in Table 1.

### 3.2. Pathogen Recognition Receptors on DCs Regulate Antigen Sensing, Uptake and Activation

DCs are mounted with PRRs from several classes comprising Toll-like receptors (TLRs), C-type lectin receptors (CLRs), scavenger receptors, Fc receptors (FcRs), retinoic acid inducible gene 1 (RIG-1)-like receptors (RLRs), nucleotide-binding oligomerization domain (NOD)-like receptors (NLRs) as well as the stimulator of interferon genes (STING) [102,109,110,111,112,113,114,115,116,117,118].

TLRs are type-I transmembrane proteins carrying an extracellular leucine-rich repeat domain (LRR) responsible for antigen recognition, while signaling is mediated by the intracellular Toll/interleukin-1R (TIR) domain [119]. In mice and men, TLR1-9 and their ligands are highly conserved, whereas TLR11-13 or TLR10 are only present in mice or men, respectively [120]. While the endosomal TLRs 3, 7, 8, 9, and 13 allow for the detection of nucleic acids (viral/bacterial RNA/DNA), the plasma membrane located TLRs 1, 2, 4, 5, 6, 10, and 11 are able to recognize conserved pathogenic sugar moieties, lipoproteins or fungal cell wall structures [121,122,123]. Additionally, the endosomal TLR11 and TLR12 detect flagellin and profilin [95,124,125]. Besides homodimerization, TLRs were also found to form heterodimers (TLR1/TLR2, TLR2/TLR6, TLR11/TLR12), thereby further increasing and fine-tuning the number of recognizable antigens [115,120,124]. A summary of pathogenic, endogenous and synthetic TLR agonists is shown in Table 2. Except for TLR3, all TLRs can signal via myeloid differentiation primary-response protein 88 (MyD88) leading to the production of pro-inflammatory cytokines and chemokines via the NF-κB pathway [114,120,126]. However, TLR3 and TLR4 can, independent of MyD88, initiate type I interferon production via a TIR-domain-containing adaptor protein inducing interferon β (TRIF)-dependent pathway, thereby fostering antiviral responses by type I interferon production [126,127]. Additionally, the TRIF dependent pathway can also lead to inflammasome activation [128].

The family of plasma membrane located CLRs can recognize self- and non-self-sugar moieties via conserved carbohydrate recognition domains (CRDs), but now it is appreciated that also side chains of proteins and glycosphingolipids can be detected [109,112,129,130]. CLRs are grouped into type I transmembrane CLRs (e.g., DEC205 and MMR) showing multiple CRDs and an extracellular N-terminus, whereas type II transmembrane CLRs carry one CRD and a cytosolic N-terminus (most other CLRs) [112,129,131]. Antigen recognition by CLRs can induce receptor-mediated endocytosis of bound material, thereby leading to antigen uptake, processing and peptide presentation by DCs [109,112]. This renders CLRs as interesting targets for antigen delivery approaches in vivo [55,56,57,62,63,109,132,133,134,135,136,137,138,139]. Additionally, some C-type lectin receptors exhibit intracellular signaling motifs either mediating activating (e.g., Dectin-1) or inhibitory/repressing (e.g., DCIR) signals via an immuno-receptor tyrosine-based activation motif (ITAM) or inhibitory motif (ITIM), respectively [129,131]. Other CLRs were described to associate with the ITAM-carrying FcεRγ-chain, which mediates signaling. These include the dendritic cell immunoactivating receptor (DCAR), Dectin-2 and macrophage-inducible C-type lectin (MINCLE) [140,141,142]. Furthermore, CLRs (as well as Scavenger receptors) were demonstrated to be involved in the detection of damage-associated molecular patterns (DAMPs) allowing for the recognition, uptake and presentation of self-derived peptides from dead or damaged cells and cell debris. These receptors include DEC205 and CLEC9A [41,143,144,145]. Interestingly, TLR stimulation was found to downregulate CLEC9A expression, thereby ensuring that self-derived peptides are only presented in an anti-inflammatory context [146]. Together with the presentation of peptides that are resulting from defective ribosomal products (DRIPs), self-peptide presentation contributes to the maintenance of peripheral T cell tolerance, but also allows for the induction of anti-tumor immune responses against self-derived antigens under pro-inflammatory conditions [147,148,149,150].

Another interesting group of receptors expressed by several immune cell types are the Fcγ receptors (FcγRs) [151]. This receptor class enables the recognition of IgG as well as IgG-induced immune complexes allowing for cell type dependent reactions including modulation of activation, endocytosis and antigen processing, antibody-dependent cellular cytotoxicity (ADCC), phagocytosis, production of pro-inflammatory cytokines and the release of vasoactive or cytotoxic substances [151,152,153,154]. On DCs in particular, FcγRs were found to mediate immune complex recognition and uptake, control maturation and activation as well as antigen presentation [53,56,102,153,154]. Mice and humans share the expression of the activating high-affinity FcγRI (CD64), which is able to bind monomeric IgG molecules, as well as the expression of the inhibitory FcγRIIB (CD32). Furthermore, mice express the activating FcγRIII (CD16) and FcγRIV (CD16.2), while the activating FcγRIIA, FcγRIIC, and FcγRIIIA are found in humans [154]. Finally, humans possess FcγRIIIB, which is unable to initiate signaling [154]. While all activating receptors mediate their signaling via an ITAM motif, either receptor intrinsic (human FcγRIIA & FcγRIIC) or via the accessory FcεRγ-chain (all other activating FcγRs), the inhibitory function of FcγRIIB is dependent on a receptor intrinsic ITIM motif [154]. While murine splenic cDC1 and cDC2 display an expression of FcγRI, FcγRIIB, FcγRIII, and FcγRIV to different extends, FcγRIIB is the only receptor expressed by murine pDCs [56]. RNA expression analyses indicate that human cDC1 and cDC2 express FcγRI, FcγRIIA, FcγRIIB, while cDC2 additionally express FcγRIIIA [30,31,33,42,89,101]. A summary of FcγR expression in murine and human cDC subsets is provided in Table 1.

Besides plasma membrane located CLRs and FcγRs, DCs are equipped with different innate intracellular sensors comprising RLRs, NLRs, inflammasomes, and STING. RLRs include RIG-I, melanoma differentiation associated protein 5 (MDA5) and laboratory of genetics and physiology 2 (LGP2) [155]. Whereas RIG-I or MDA5 are in charge for sensing either short or long double stranded RNA (dsRNA) leading to subsequent production of type I IFNs and pro-inflammatory cytokines via IRFs and NF-κB, LGP2 is thought to regulate other RLRs [110,155]. Even though the role of LGP2 during RLR dependent anti-viral responses is differentially discussed in the literature, a recent study suggests that LGP2 boosts MDA5 dependent responses, while ameliorating RIG-I pathways partially by coordinating post transcriptional RNA silencing programs [156,157]. In addition to dsRNA, RNA polymerase III activity enables the RIG-I dependent sensing of viral dsDNA following translation to a poly(dA:dT) dsRNA [158]. Interestingly, RIG-I and MDA5 exhibited a higher abundance in murine cDC2 [88]. Compared to cDC1, infection with ssRNA viruses, such as Sendai virus or influenza A virus, led to a stronger RLR-dependent type I IFN response in cDC2, even though both subsets were similarly infected [88]. Together with the presence of cytoplasmic NLR family members, the pronounced expression of RLRs may render cDC2 as rapid sensors for cytoplasmic pathogens [88]. An earlier study by Wilson et al. suggested that the cross-presentation ability of exogenously acquired material was downregulated following TLR ligand recognition, while presentation of endogeneous material remained intact [159]. Since cDC1 will likely encounter and ingest infected and/or dead cell material leading to activation of endosomal TLRs, antigen uptake and thus cross-presentation on MHC I might be prolonged in the absence of cytoplasmic viral sensors in cDC1. This might potentially open a timeframe for kick-starting CTL responses before the cross-presentation machinery and the presentation of viral peptides is downregulated, even if cDC1 are directly infected [88,159]. Thus, treatment regimen first delivering antigens for cross-presentation to cDC1 and stimulating type I IFN production by cDC2 followed by complete activation of cDC1 might be a promising approach to boost the early phase of CTL induction. Finally, polyinosinic-polycytidylic acid (poly(I:C)) was validated as MDA5 ligand [160]. Hence, poly(I:C) could in principle directly activate cDC1 and cDC2 via their putatively specialized expression of TLR3 or MDA5, respectively. This activation was dependent on type I IFN pathways increasing the immunogenicity and presentation capacity of DCs [161]. Of note, while poly(I:C) application only allowed for the induction of cDC1-dependent cytotoxic CD8^+^ T cell responses, cDC2 could not induce a cytotoxic CD8^+^ T cell response upon poly(I:C) stimulation, if cDC1 were missing. However, a direct cDC2 stimulation via R848 (TLR7 ligand) allowed for the induction of a cytotoxic CD8^+^ T cell response [162].

The family of NLRs encompasses several members sharing characteristic structural motifs including a nucleotide-binding NACHT domain enabling oligomerization, an N-terminal effector domain mediating downstream effector functions and a C-terminal leucin-rich repeat domain (LRR) in charge of pattern recognition [163,164]. Depending on their N-terminal domain composition, NLRs are subdivided into four different families including 22 members in humans, namely NLRA (CIITA), NLRB (NAIP), NLRC (including NOD-1 and 2), and NLRPs [164]. NOD-1 and -2 (alias NLRC1 and 2) are classical examples for pattern recognition receptors. Following recognition of peptidoglycans of gram-negative, but in particular of gram-positive bacteria, NODs are major inducers of the downstream NF-κB [116,165,166]. In DCs, administration of antigens and NOD agonists fostered Th2 cell polarization in an OX-40 ligand dependent manner, even though the capacity to induce other co-stimulatory molecules on DCs was low [167,168]. This is in line with the observation that NOD-1 is primarily expressed by murine cDC2 [88]. Thus, application of NOD agonists may serve as interesting strategy for the modulation of Th1-driven autoimmune conditions. However, this might not render NOD agonists as ideal adjuvant candidates for tumor therapy. Nevertheless, simultaneous integration of parallel NOD and TLR signaling is thought to be important for Th1, Th2 and Th17 cell induction [169]. Finally, NODs were shown to foster targeting of intracellular bacteria into the autophagy pathway, while downregulating inflammasome activity [116,170,171,172,173,174].

Besides NOD-1 and -2, inflammasome activating NLRs have gained attention within the last decade. In particular, mechanisms associated with NOD-, LRR- and pyrin domain-containing protein 3 (NLRP3) have been excessively studied even though NLRs from other subfamilies, such as NLRC4, are able to assemble inflammasomes following challenge with environmental irritants [118]. While this review will focus on the well-characterized NLRP3, information on other NLRs able to trigger inflammasome activation is nicely provided elsewhere [175].

Inflammasomes are multi-protein complexes and their assembly and function is dependent on two signals. First, environmental sensing of PAMPs, DAMPs and/or cytokines leads to the NF-κB dependent transcription of inflammasome building blocks and the target molecules pro-IL-1β and pro-IL-18 [176,177,178]. In addition, environmental sensing was described to introduce stabilizing or destabilizing post-transcriptional modifications to NLRP3 in a context-dependent manner [118]. This priming procedure of target cells hinders premature inflammasome activation without respective stimuli, thus licensing cells for the second step: the inflammasome activation. So far, an array of inflammasome activators has been identified as reviewed by Swanson et al. [118]. Although, these activators stem from various classes including self- and non-self-derived DAMPs or PAMPs utilizing different cellular pathways, they are unified by their ability to induce cellular stress. Cellular products induced by irritant activated emergency pathways finally lead to NLRP3 activation and oligomerization allowing for assembly of the multiprotein inflammasome complex. This complex encompasses the sensor (NLRP3), the adaptor protein ASC (PYCARD) in charge of caspase recruitment, and the effector protein caspase-1 [118,179,180,181]. In the case of NLRP3 induced inflammasomes, NIMA-related kinase 7 (NEK7) was also described to be essential for NLRP3-dependent inflammasome assembly [182,183]. Finally, activated caspase-1 can cleave pro-IL-1β, pro-IL-18 and gasdermin D to functional effector molecules. With respect to DCs, TLR4 stimulation by LPS was shown to be sufficient for inflammasome assembly without initial priming leading to a rapid DC response, even though the exact mechanism needs to be clarified [184]. Beneath the canonical pathway of inflammasome activation, cytosolic LPS detection via caspase 4 and 5 in humans or 11 in mice can lead to non-canonical inflammasome activation also fostering pro-IL-1β, pro-IL-18 and gasdermin D cleavage [185,186,187].

In addition to the production of inflammatory cytokines, inflammasome activation can induce a form of inflammatory cell death referred to as pyroptosis. Following gasdermin D cleavage, activated gasdermin D integrates as multimer into the membrane leading to pore formation and pyroptotic cell death, thereby fostering rapid IL-1β release [187,188,189,190]. In the case of DCs, induction of pyroptosis was dependent on the inflammasome ligand. While LPS in combination with oxidized phospholipids (oxPAPC) facilitated IL-1β release, only LPS in combination with ATP and not oxPAPC triggered pyroptosis [191]. This is in line with a study providing evidence that IL-1β secretion by non-pyroptotic cells occurs without the involvement of gasdermin D [192]. Further, oxPAPC alone did not induce IL-1β release [191]. Thus, specific combinations of DC inflammasome priming and activating agents may allow for inflammasome activation without negatively affecting DC viability and thereby the T cell priming potential [193]. The combination of efficient pro-inflammatory cytokine production and release of IL-1β while maintaining viability is called hyperactivation. Additionally, ATP released by dying tumor cells links cell death with inflammasome activation [194]. In the context of chemotherapy, a study provided evidence that the NLRP3 inflammasome including caspase-1 are important for the induction of tumor-specific IFNγ producing helper CD4^+^ T cells, since Th1 priming failed in NLRP3 or caspase-1 deficient mice [194]. As the concept of DC hyperactivation is only settled for bone-marrow derived DCs, it remains elusive, if and to which extend primary DCs can undergo hyperactivation. However, by combining protein and expression profiling in human cDC1, cDC2, and pDCs, superior expression of several inflammasome pathway components and the effector cytokines IL-1β and IL-18 was demonstrated in cDC2 [92]. cDC1 only displayed high levels of absent in melanoma 2 (AIM2) [92]. pDCs did not express considerable amounts of any of the investigated inflammasome pathway components rendering pDCs inert and cDC2 highly responsive to inflammasome activation [92]. This fact was underlined by the high IL-1β release from cDC2 and its absence in pDCs following TLR7/8 priming with R848 and stimulation with ATP [92].

Since IL-1β is a key cytokine utilized by DCs to polarize Th17 responses (discussed in Section 4.2.3), the activation of the inflammasome could lead to an unwanted Th17 differentiation of naïve CD4^+^ T cells. On the other hand, cytokines released during hyperactivation of DCs without the induction of pyroptosis could shift gears in the induction of T cell responses by DCs. Therefore, the application of inflammasome modulators might represent an attractive possibility to modify the balance of different T helper cell responses (Th1 vs. Th2 vs. Th17) or utilize the described plasticity of e.g., Th17 cells for tumor therapy [195,196,197,198]. Molecular mechanism and cellular pathways dealing with inflammasome priming, activation, assembly and downstream effector modules have recently been thoroughly summarized elsewhere [118,199,200].

The stimulator of interferon genes (STING; alias MITA, ERIS, MPYS, or TMEM173) was recognized as another powerful intracellular DNA sensor enabling recognition of cyclic GMP-AMP (cGAMP) derived from cytosolic ssDNA and dsDNA. DNA sources include viral and self-DNA or cyclic dinucleotides (CDNs) produced by intracellular bacteria [111,201,202,203,204,205,206,207,208,209]. Following intracellular DNA sensing, the cyclic GMP-AMP synthase (cGAS) produces cGAMP, which is subsequently recognized by STING, from cytosolic DNA in the presence of ATP and GTP [206,210,211]. STING is expressed by multiple cell types including T cells, macrophages, and DCs and mainly resides in the membrane of the endoplasmic reticulum [203,204]. Following ligand recognition, STING dimerizes, thereby allowing for downstream signaling via IRF3 and NF-κB fostering strong induction of innate immune defense genes including type I IFNs [203,204,207,209,212]. By these mechanisms, cytosolic DNA indicating DNA damage is serving as potent DAMP.

An early study demonstrated that mice deficient for MyD88 are less susceptible to carcinogenesis in the skin following application of the DNA damaging agent DMBA, thus implying a direct role for pro-inflammatory cytokine production in inflammation-induced skin tumors [213,214]. However, the initial trigger leading to MyD88-dependent cytokine production was not identified. In a later study, DMBA was found to cause DNA leakage into the cytosol, thereby boosting carcinogenesis in the skin in a STING-dependent fashion [215]. Since the STING-dependent immune response was not affected in MyD88-deficient cells, this may imply that products derived from the STING pathway, in particular pro-inflammatory cytokines, are the initial MyD88 trigger or that STING-dependent carcinogenesis functions independently of MyD88 [216]. Furthermore, STING was described to induce tolerogenic responses including expression of indoleamine-2,3-dioxygenase (IDO) in DCs fostering IL-10 production, myeloid suppressor cell recruitment and tumor progression of low-antigenic tumors [217,218]. However, the roles of MyD88 and STING may be dependent on the micromilieu, since the majority of models provide evidence for potent anti-tumor effects of MyD88 and STING. For instance, STING and MyD88 were drivers for prevention of colitis-associated cancer induced by DNA damaging agents by kick-starting tissue repair pathways and the tumor suppressive cytokine IL-22BP [216,219]. Finally, tumor-derived cGAMP is key for anti-tumor responses by NK cells, thereby partly determining tumor immunogenicity [220]. Thus, STING activity may be essential to forestall the early loss of immunosurveillance of DNA damaged cells [216,219,221,222].

STING profoundly supports both, the spontaneous and therapeutic induction of cytotoxic effector modules. In particular, type I IFN production by cDC1 constitutes an important T cell driver during cross-presentation and priming of CTL responses [223,224,225]. Thus, sensing of cytosolic DNA following engulfment of dead or necrotic tumor cells by highly phagocytic cells, such as DCs, can drive sterile inflammation via type I IFN induction, thereby enabling efficient anti-tumor responses [225,226,227,228]. In this respect, STING may help to overcome the issue of immunologically silenced cell death within tumor tissues. To tackle the unwanted inhibition of productive immune responses to such cells, the development of antagonists for STING-suppressive pathways including caspase-9 or caspase 3/7 inhibitors or by interfering with the rapid regulatory STING turnover, might circumvent immunologically silenced apoptosis and might enhance anti-tumor responses [229,230]. Overall, STING deficiency was associated with a failure of T cell priming potentially caused by a lack of co-stimulation during the induction of anti-tumor responses [225]. The importance of STING for the onset of anti-tumor immune responses also becomes evident during therapeutic intervention, since utilizing synthetic CDN derivate-based vaccines, which are used by cGAS for the production of the STING ligand cGAMP, in different poorly immunogenic tumor models induced tumor regression in contrast to PD-1 monotherapy [223]. In general, the therapeutic potential of STING or cGAS ligands was demonstrated in various studies highlighting the therapeutic potential of this strategy [223,224,231,232,233,234]. Furthermore, this data suggests that STING may be the immunologic hub driving effective adaptive immune responses and synergizing with radiation-, chemo- or anti-cancer/mitotic drug therapy [225,227,235,236,237,238]. Finally, the STING pathway is also employed by T cells for retrograde signaling and priming of DCs to induce IFN production [239]. Thus, targeted delivery of tumor-specific antigens and STING agonists to DCs may represent a promising approach for overcoming immune-regulatory properties of the STING pathway and to enhance cross-presentation via type I IFN induction.

The structure, mode-of-action, and importance of STING in pathogen defense, autoimmunity and cancer including therapeutic approaches have recently been summarized in multiple terrific reviews [110,111,202,240,241,242,243].

**Table 2:** Summary of pathogenic, endogenous and synthetic ligands for pattern recognition receptors.

**TLR Ligands**				
**Receptor**	**Organism**	**Ligand Class**	**Molecules**	**References**
TLR2 (TLR1/TLR2; TLR2/TLR6)	M/H	Pathogenic	Lipoptroteins; Triacetylated lipopeptides; human β-defensin 3 (BD-3); Glycosylphosphatidylinositol (GPI); Zymosan; Peptidoglycan; LPS; Lipoproteine/-peptide; Glycolipide; MALP-2; Diacetylierte Lipopeptide; LTA; Zymosan	[244,245,246,247] [248,249,250,251,252,253,254,255,256,257,258]
		Endogenous	Endoplasmin; Hsp60; Hsp70; Human cardiac myosin; Urate crystals; Hyaluronan	[259,260,261,262,263,264]
		Synthetic	FSL-1, synthetic lipopeptides & lipoprotein analogs, triacetylated lipopeptides e.g., Pam2CSK4, Pam3CSK4, or synthetic beta-defensin 3, MALP2	[244,245,258], [265,266,267,268]
TLR3	M/H	Pathogenic	dsRNA	[269,270,271]
		Endogenous	mRNA	[270]
		Synthetic	poly(I:C); poly(A:U)	[258,272,273,274]
TLR4 (MD-2; CD14)	M/H	Pathogenic	Lipopolysaccharid (LPS; Gram-); Lipoteichoic acid (LTA; Gram+)	[256,275,276]
		Endogenous	β-defensin 2; Fibronectin; Fibrinogen; Hyaluronan; Surfactant protein A; Urate crystals; OxPAPC; Hsp72; Hsp70; Hsp60; HMGB1; Endoplasmin	[262,264,277,278,279,280,281,282], [259,260,283,284]
		Synthetic	Glycan-based agonists; Monophosphoryl Lipid A (MPL); pyrimido[5,4-b]-indoles; LPS	[268,285,286,287]
TLR5	M/H	Pathogenic	Flagellin	[248,288]
		Endogenous	Unknown	
		Synthetic	Flagellin	[289,290]
TLR7	M/H	Pathogenic	ssRNA	[291,292,293]
		Endogenous	RNA; siRNA	[294,295]
		Synthetic	Imiquimod (R837); Resiquimod (R848); Gardiquimod; Loxoribine	[268,274,296,297,298,299,300,301]
TLR8	M/H	Pathogenic	TLR7 antagonist (mouse); ssRNA (human)	[292,299,302,303]
		Endogenous	Human cardiac myosin; siRNA	[295,304]
		Synthetic	Imiquimod (R837); Resiquimod (R848); Gardiquimod; TL8-506	[268,274,296,297,298,299,300,301]
TLR9	M/H	Pathogenic	Unmethylated CpG DNA; dsDNA	[258,269,305,306]
		Endogenous	DNA; HMGB1	[307,308,309]
		Synthetic	Diverse synthetic CpG-oligonucleotides (CpG-ODNs)	[268,274,310,311,312]
TLR10	H	Pathogenic	Non functional in mice (viral insertion); TLR2 antagonist; HIV-1 gp41	[120,313,314]
		Endogenous	Unknown	
		Synthetic	Unknown	
TLR11	M	Pathogenic	Profilin	[95,120,315]
		Endogenous	Unknown	
		Synthetic	Unknown	
TLR12	M	Pathogenic	Profilin	[120,315,316]
		Endogenous	Unknown	
		Synthetic	Unknown	
TLR13	M	Pathogenic	Bacterial 23S rRNA	[120,123]
		Endogenous	Unknown	
		Synthetic	Unknown	
CD180	M/H	Pathogenic	Unknown	
		Endogenous	Negative regulator of TLR7 & TLR9	[317]
		Synthetic	Unknown	
**STING ligands**	**Organism**	**Ligand Class**	**Molecules**	**Literature**
**Receptor**				
STING	M/H	Pathogenic	Bacterial cyclic di-nucleotides and cGAMP; viral DNA	[111,206,210,211]
		Endogenous	Self-DNA e.g., dead cells, DNA leaking into the cytosol following stress or tumor DNA; tumor cGAMP	[220,225,227,228]
		Synthetic	Cyclic dinucleotides e.g., 2′-3′-cGAMP; c-di-AMP; di-amidobenzimidazole	[301,318]
**RLR ligands**	**Organism**	**Ligand Class**	**Molecules**	**Literature**
**Receptor**				
RIG-I	M/H	Pathogenic	Paramyxoviridae, Rhabdoviridae & Orthomyxoviridae; short dsRNA; dsDNA in cooperation with RNA Polymerase III; 5′-phosphorylated ssRNAs	[110,155,158,160,319]
		Endogenous	Endogeneous RNAs e.g., LINE1	[320,321]
		Synthetic	poly(dA:dT); tri-phosphorylated 5′ stem-loop RNAs	[158,322,323]
MDA-5	M/H	Pathogenic	Picornaviridae; long dsRNA; ssRNA; dsDNA; NAB2; rb-dsRNA	[110,155,160,322,324]
		Endogenous	Endogeneous RNAs e.g., mitochondrial RNA and retroelement transcripts	[320,325,326,327]
		Synthetic	poly(I:C)	[160,322]

M = murine, H = human.

## 4. DCs Orchestrate T Cell-Driven Immunity

### 4.1. The Potential of DC:T Cell-Based Vaccines and the Importance of CD4^+^ T Cell Help for Cancer Immunotherapy

Although most of our current vaccination approaches rely on the effective induction of high-affinity, class-switched antibodies, T cells are good candidates to deal with viral infections and malignant diseases. While CTLs are able to directly recognize and kill target cells following pMHC-I recognition, today it is appreciated that helper CD4^+^ T cells are powerful players in DC vaccination approaches. The reason for this is the importance of CD4^+^ T cell help for various phases of antigen-specific cytotoxic CD8^+^ T cell responses, such as initial priming, memory formation and termination of an immune response [328,329,330,331,332]. This is underpinned by the observation that clinical treatment regimens aiming for induction of tumor-specific helper CD4^+^ T cells led to durable anti-cancer responses [333,334]. Additionally, the existence of lytic CD4^+^ T cells able to kill target cells following pMHC-II recognition in a contact dependent fashion via granzyme B and perforin has been described in the past [335,336,337]. Such lytic CD4^+^ T cells were able to eradicate solid tumors in murine melanoma models [338,339].

After emerging from the thymus, naïve CD8^+^ T cells must undergo a process termed licensing. Utilizing different models of viral infection in mice, intravital microscopy demonstrated that cytotoxic CD8^+^ and helper CD4^+^ T cells assemble around resident XCR1^+^ cross-presenting cDC1 following initial contact of both T cell populations with different migratory DCs in a spatially separated manner [340,341]. It is assumed that following antigen transfer, resident XCR1^+^ cDC1 concomitantly present pMHC-I and pMHC-II complexes allowing the formation of a three-cell type platform endowing cytotoxic CD8^+^ T cells with the license to kill [332,340,341,342,343,344]. This platform enables the translation of helper CD4^+^ T cell signals (CD40:CD40L axis) by cDC1 to cytotoxic CD8^+^ T cells (e.g., via the CD70:CD27 axis and/or IL-15 secretion by licensed DCs) since DCs bring both populations into close proximity by chemokine baiting [330,343,345,346,347,348,349]. As the effective priming of cytotoxic CD8^+^ T cell immunity requires mounted helper CD4^+^ T cells, these cells should be induced earlier during the induction of an immune response. This concept is supported by the finding that cDC2 and helper CD4^+^ T cells accumulate in the periphery of lymph nodes at the sites of antigen entry, while cDC1 are located in the center [350,351]. Disruption of this specialized localization patterns led to lack of CD4^+^ T cell help ultimately impairing the formation of cytotoxic CD8^+^ T cell memory [350]. Additionally, by utilizing a moDC-based in vitro system, a study by Hoyer et al. demonstrated that simultaneous encounter of helper CD4^+^ T cells and cytotoxic CD8^+^ T cells with DCs is required for optimal cytotoxic CD8^+^ T cell priming [352]. Considering that sequential interaction of helper CD4^+^ T cells and cytotoxic CD8^+^ T cells with DCs were inefficient in inducing cytotoxic CD8^+^ T cell expansion, these findings indicate that DC licensing by helper CD4^+^ T cells is transient and/or close proximity between CD4^+^ and CD8^+^ T cells is required. This proximity allows in turn for an efficient direct communication of helper CD4^+^ T cells and cytotoxic CD8^+^ T cells either via surface receptors or cytokine secretion. For instance, besides binding to CD40 on DCs, helper CD4^+^ T cell-derived IL-2 during priming was essential for the effective induction of cytotoxic CD8^+^ T cell responses to non-inflammatory antigens and for cytotoxic CD8^+^ T cell memory formation [353,354]. While T cell help during priming was directly translated into a highly efficient cytotoxic CD8^+^ T cell effector phenotype including the migratory potential and downregulation of inhibitory cytotoxic CD8^+^ T cell receptors, the transcriptome of unhelped cytotoxic CD8^+^ T cells resembled exhausted T cells. This led to impaired anti-tumor immune responses [345,355]. Besides, helper CD4^+^ T cells were described to mediate entry of cytotoxic CD8^+^ T cells into tissues, thus potentially contributing to cytotoxic CD8^+^ T cell recruitment to tumor sites [356]. Furthermore, a cross-tissue study by Spitzer et al. demonstrated that tumor eradication required systemic immune responses, as peripheral helper CD4^+^ T cells mediated the protection against new tumors [357]. The systemic response could already be detected in murine blood, and a similar T cell population was expanded in melanoma patients responding to CTLA-4 blocking immunotherapy [357]. Additionally, this study demonstrated that the observed systemic remodeling extended to the bone marrow [357]. There, the generation of matured cells, including B cells, CD4^+^ as well as CD8^+^ T cells, macrophages, and pDCs, was enhanced, without significant changes in the frequency of their respective progenitor populations. Thus, this study suggests that (I) systemic induction of helper CD4^+^ T cells and (II) reprogramming of hematopoiesis and manipulation of the precursor pool might be essential for effective cancer therapy and that (III) analysis of the blood immune cell pool might serve as biomarker for the efficacy of cancer treatments [357].

Concomitant to the induction of better helper CD4^+^ T cells, the manipulation of XCR1^+^ cDC1 might be beneficial for releasing the true power of cytotoxic CD8^+^ T cell responses as we will discuss later. Furthermore, circumventing the generation and restricting the activity of T_reg_ cells suppressing anti-tumor effector cells is of major importance [358]. While these cells are designed to maintain immune homeostasis by controlling autoreactive cells and the inflammatory milieu, tumors can exploit T_reg_ cell intrinsic functional features to facilitate tumor progression [359]. These functions include suppression of DCs by T_reg_ cells via transendocytosis or blocking of CD80 and CD86 by CTLA-4, thereby destabilizing the DC:CTL interaction and/or by simple binding competition for IL-2, thus promoting T cell quiescence [331,360]. However, T_reg_ cells seem to constitute a double-edged sword during the orchestration of cytotoxic CD8^+^ T cell responses as T_reg_ cells not only function to negatively influence the induction of cytotoxic CD8^+^ T cells, but also contribute to memory formation [331,361]. The role of helper CD4^+^ T cells for cytotoxic CD8^+^ T cell responses and memory formation are thoroughly summarized elsewhere [329,331].

### 4.2. The Priming of Naïve T Cells by DCs Is Regulated by Three Signals

**Figure 1:**
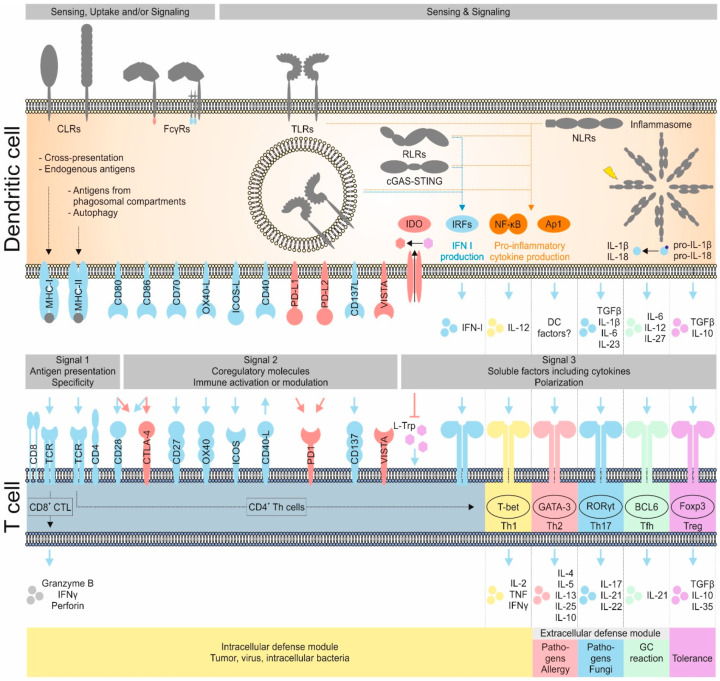
Translation of environmental sensing by dendritic cells to defined T cell effector modules occurs at the molecular interface of the immunological DC:T cell synapse. Dendritic cells (DCs) are equipped with an array of sensory receptors allowing for sampling of the surrounding and detection of endogenous (DAMPs) and pathogenic material (PAMPs) enabling antigen uptake and/or signaling. While CLRs and FcγRs are major contributors to antigen uptake, other receptors, including TLRs, RIGs, cGAS-STING, and NLRs initiate signaling following antigen recognition. While TLRs located in the plasma membrane allow for sensing of a multitude of different exogenous, pathogenic ligands mainly derived from bacteria or fungi, endosomal TLRs and cytoplasmic RIGs track nucleic acids of viral origin. NOD proteins of the NLR family act in the recognition and defense against intracellular bacteria by recognizing cytosolic peptidoglycans. On the other hand, the cGAS-STING is specialized in the detection of cellular DNA acquired from necrotic cell material. Ligand recognition by TLRs and NODs in the plasma membrane leads to the production of pro-inflammatory cytokines via the transcription factors NF-κB and AP-1, whereas endosomal TLRs, RIG and cGAS-STING allow for simultaneous induction of pro-inflammatory cytokines or type I IFN production via NF-κB and AP1 or IRFs, respectively. The multiprotein-complexes called inflammasomes are ascertained as cytoplasmic sensors for a variety of stimuli including DAMPs, such as urate crystals, and PAMPs leading to caspase-1-dependent release of IL-1β. Antigen uptake and signaling directly translates the environmental sensing of DCs into distinct T cell effector modules and functional states. This translation occurs at the molecular interface of DCs and T cells during T cell priming and is dictated by three signals. While presentation of peptides in the context of MHC-I, either derived from DRiPs or by cross-presentation following uptake of exogenous material, allows for the induction of cytotoxic CD8^+^ T cell responses, presentation of exogenously acquired material on MHC-II molecules or intracellular peptides derived from autophagy enable the priming of helper CD4^+^ T cell responses. Thereby, antigen presentation, called signal 1, provides both, specificity and separation into cytotoxic CD8^+^ and helper CD4^+^ T cell responses. Signal 2 and signal 3 directly result from the signaling processes initiated in DCs following sensing of environmental cues, whereas signal 2 comprises the direct interaction of DC and T cell co-regulatory molecules determining immune activation or modulation, signal 3, mainly consisting of soluble DC-derived factors, orchestrates the polarization of naïve T cells into distinct effector populations. However, it should be recognized that signal 2 (e.g., CD70:CD27) or signal 3 (e.g., IFN-I or L-Trp) can contribute to polarization or immune modulation, respectively. In general, distinct DC cytokine portfolios lead to the polarization of naïve T cells to Th1 (dependent on the transcription factor T-bet), Th17 (dependent on RORγt), Tfh (dependent on BCL6), or T_reg_ cells (dependent on FoxP3). Up to date, no DC-derived soluble factors directly promoting Th2 differentiation (dependent on GATA-3) have been identified. Polarized T cells subsequently contribute to different effector modules including the defense against intracellular pathogens and tumors mediated by cytotoxic CD8^+^ T cells and Th1 cells. Th2, Th17 and Tfh cells are specialized for fighting extracellular pathogens including bacteria, fungi and helminths. T_reg_ cells are designed to maintain peripheral tolerance. This figure does not include the complete repertoire of molecules cytokines or signaling pathways acting at the interface of DC and T cell communication. DC = dendritic cell; CLR = C-type lectin receptor; FcγR = Fc gamma receptor; TLR = Toll-like receptor; RLR = RIG-I like receptor; cGAS-STING = cGAMP synthase-stimulator of interferon genes; NOD = nucleotide-binding oligomerization domain proteins; NLR = NOD-like receptor; DRiPs = defective ribosomal products; IL = interleukin, IFN = interferon; MHC = major histocompatibility complex; CD = cluster of differentiation; IDO = indoleamine-2,3-dioxygenase; IRF = interferon regulatory factor; NF-κB = nuclear factor kappa-light-chain-enhancer of activated B cells; AP1 = activator protein 1; TGFβ = tumor growth factor beta; TCR = T cell receptor; CTLA4 = cytotoxic T lymphocyte antigen 4 (CD152); PD1 = programmed cell death protein 1; PD-L1 or 2 = programmed cell death protein 1 ligand 1 or 2; VISTA = V-domain immunoglobulin suppressor of T cell activation; ICOS = inducible T cell co-stimulator; ICOS-L = inducible T cell co-stimulator ligand; CTL = Cytotoxic T lymphocyte; Th = T helper cell; T-bet = T-box expressed in T cells; GATA-3 = GATA binding protein 3; BCL6 = B cell lymphoma 6; FoxP3 = Forkhead box P3; L-Trp = L-Tryptophan.

In secondary lymphoid organs, DCs present pMHC complexes to prime naïve T cells [26]. This priming procedure includes three regulatory signals: the presentation of pMHC to the T cell receptor (TCR) (signal 1), co-regulatory signals via activating or inhibitory surface molecules (signal 2), and the secretion of soluble mediators (signal 3) [362,363,364]. Within this process, the combined phenotype of signal 2 and 3 directly results from the signaling cascades downstream of environmental monitoring by DCs, thereby translating the microenvironment or select adjuvants into distinct T cell effector modules [104]. The complete process of environmental sensing and its translation into distinct T cell effector modules at the interface of DC:T cell communication is summarized in Figure 1.

#### 4.2.1. Signal 1: pMHC Recognition by the TCR

Peptides presented on MHC molecules are recognized by antigen-specific T cells following a two-step lock-and-key model [365,366]. While MHC is able to generate a short initial contact independent of the TCR specificity, the interaction is stabilized depending on the peptide structure, while for overall interaction the amount of presented antigenic peptides is thought to be critical [367,368,369]. The recognition of pMHC-I complexes requires the TCR and the CD8α co-receptor expressed on CD8^+^ T cells, whereas pMHC-II complex recognition relies on the simultaneous action of the TCR and the co-receptor CD4 expressed by CD4^+^ T cells. Since the TCR co-receptor expression is restricted to either CD8^+^ or CD4^+^ T cells, while cDC1 and cDC2 express MHC-I and MHC-II, both DC populations are in principle able to prime CD8^+^ and CD4^+^ T cell responses. However, cDC1 excel in their capacity to cross-prime cytotoxic CD8^+^ T cells with extracellularly acquired antigens and Th1 cells responses, while cDC2 are major contributors to Th2 and Th17 responses and their transcriptional profiles highlight the specialization of cDC1 and cDC2 for the MHC-I and MHC-II machinery, respectively [39,54,55,56,370,371]. How cDCs orchestrate T cell activation, suppression and polarization is summarized in the following sections. Owing to the plethora of regulatory molecules acting in DC:T cell communication, this review will focus on the functional outcome of different regulatory axes under normal circumstances and during cancer therapy. Nevertheless, we provide recent reviews discussing molecular features, such as molecular structure and detailed signaling pathways.

#### 4.2.2. Signal 2: Co-Regulatory Surface Molecules Define the Functional Phenotype of Primed T Cells

Following engagement of the TCR and initiation of signaling, co-stimulatory or -modulatory surface receptors further manipulate and fine-tune the T cell responses induced. Therefore, DCs and T cells express an array of regulatory surface proteins acting in DC:T cell cross-talk. On the DC side, the activation markers CD80 (B7.1) and CD86 (B7.2), which are dynamically regulated following the sensing of environmental cues, can directly interact with the CD4^+^ and CD8^+^ T cell costimulatory molecule CD28 or the inhibitory receptor CTLA4 (CD152), thereby promoting or inhibiting TCR signaling activity, respectively [372,373,374,375,376]. Interestingly, CD80 homodimers bind to CTLA-4 with a higher avidity than to CD28, while the monomer CD86 recognizes CD28 with the lowest affinity [377,378,379]. Thereby, CTLA-4 can counteract CD28 by simple ligand binding competition. Whereas CD28 is constitutively expressed in the T cell plasma membrane, CTLA-4 mainly localizes in intracellular vesicles of FoxP3^+^ T_reg_ cells or activated T cells but is rapidly recruited to the cell surface following Calcium influx induced by TCR signaling [380,381,382]. In contrast to CD28, ligation of CTLA-4 counteracts tyrosine kinase dependent TCR signaling by associating with the tyrosine phosphatase SYP [374]. Additionally, CTLA-4 regulates the amount of CD80 and CD86 on APCs by trans-endocytosis, thereby leading to removal of CD80 and CD86 from the APC surface and subsequent degradation inside of CTLA-4^+^ cells. This indicates that the parallel manipulation of DCs and CTLA-4^+^ T cells might be important for the effective induction of T cell immune responses [383].

Eradication of tumors by releasing the brakes of T cell immunity via checkpoint blockade of CTLA-4 utilizing monoclonal antibodies was demonstrated very early [384]. While the major model proposes that application of CTLA-4 checkpoint inhibitors mainly works by blocking the interaction of CTLA-4 on effector T cells allowing for their correct priming and by suppressing the activity of T_reg_ cells, some studies suggest that the function of CTLA-4 blocking antibodies relies on the fragment crystallizable (Fc) part of the IgG molecule. They consequently link the respective effector functions to the depletion of T_reg_ cells within the tumor microenvironment via antibody-dependent cellular cytotoxicity mediated by FcγR expressing macrophages [385,386,387,388,389]. However, releasing the brakes bears the danger of the development of autoimmunity [390]. In particular, checkpoint blockade of CTLA-4 was associated with a broadening of the T cell receptor repertoire due to lack of negative selection during T cell priming in the thymus [391]. Additionally, the microbiome is thought to contribute to the clinical outcome of CTLA-4 blockade since efficient responses required the presence of specific bacterial species [392]. Very recently, Zenke et al. provided a model for how a CD28 and CTLA-4 antagonistic feedback-loop can decide over productive CTL immunity or inhibition [393]. There, the T cells’ activation threshold is set by the number of reacting T cells. While a low density of T cells was efficiently primed by DCs, a high density of T cells led to a decreased autocrine IL-2 production in cell culture systems as well as LCMV infection models [393]. This was dependent on the interaction of T cell intrinsic CD80 and CD86, which was potentially acquired from APCs via trogocytosis, with primarily CD28 or CTLA-4 under low- or high-density conditions, respectively [393,394,395]. Thus, the term strength-in-numbers may not apply for T cell priming.

Another important co-stimulatory molecule on CD4^+^ and CD8^+^ T cells in mice and humans is inducible T cell co-simulator (ICOS; CD278). ICOS is upregulated following TCR and/or CD28 engagement, while ICOS-Ligand (ICOS-L) is expressed on activated APCs [396,397,398,399,400]. Comparable to other co-regulatory molecules, stimulation of ICOS via ICOS-L was described to contribute to different aspects of T cell function including proliferation, regulatory function and polarization of CD4^+^ T cells into Th1, Th2 or Tfh cells in a context dependent fashion as summarized elsewhere [401]. Interaction of ICOS-L with ICOS led to downregulation of ICOS-L expression, thereby directly regulating the activating potential of ICOS-L [402]. Besides the function of ICOS on the T cell side, Hedl et al. demonstrated by utilizing human moDCs that reverse signaling via ICOS-L is able to potentiate PRR signaling in APCs [403]. However, ICOS and ICOS-L are not only associated with beneficial stimulatory effects, as ICOS was important for T_reg_ cell maintenance and function [404,405]. These results are in line with studies from human cancer patients revealing that ICOS-L expressing pDCs fostered a regulatory T cell response, thereby favoring disease progression [397,406]. Interestingly, the simultaneous application of ICOS agonists together with checkpoint inhibitors was protective in several murine cancer models [398,407]. Moreover, the single application of αCTLA-4 checkpoint inhibitors increased the frequency of antigen-specific IFNγ producing ICOS^+^ CD4^+^ T cells correlating with a better therapeutic outcome in human cancer patients [408,409,410,411]. This indicates that ICOS expression may be a suitable biomarker during αCTLA-4 therapy. Finally, ICOS-dependent signaling was important during CTLA-4 blockade for the increase of T-bet expression, thereby boosting Th1 dependent anti-tumor immunity [412].

4-1BB (CD137 or TNFRSF9) is another co-regulatory molecule on T cells, while its ligand 4-1BB-L (CD137-L) is expressed on activated APCs. Triggering of 4-1BB on CD8^+^ and CD4^+^ T cells fostered IL-2 production in a CD28-independent manner [413]. Engagement of 4-1BB paralleled by blocking of programmed cell death protein 1 (PD-1) synergized in reducing the viral load in a model of LCMV infection in a cytotoxic CD8^+^ T cell dependent manner [414]. However, this synergy was dose-dependent as dual high dose application led to cytotoxic CD8^+^ T cell apoptosis following initial expansion accompanied by a loss of virus control [414].

Glucocorticoid-induced tumor necrosis factor receptor (GITR) is constitutively expressed on murine and human effector CD4^+^ and CD8^+^ T cells at low levels showing rapid upregulation following activation. Of note, this receptor is highly expressed on T_reg_ cells in both species [415]. However, its expression is not restricted to T cells, as murine B cells, NK cells, NKT cells, granulocytes as well as human macrophages are also positive for GITR [415].

On the other hand, GITR ligand (GITR-L) is expressed by DCs, macrophages, monocytes, and endothelial cells in mice and men [415,416,417,418]. Additionally, its expression has been observed on murine monocytes, B cells, osteoclasts and microglia [419,420,421,422,423]. On the DC side, blockade of GITR-L downregulated the migratory capacity of cutaneous CD11c^+^ DCs, potentially via a CCR7-dependent mechanism [417]. On T cells, GITR ligation by agonistic antibodies or a soluble GITR-L was able to overcome T_reg_ cell mediated suppression of other T effector cells [423,424,425]. To avoid GITR-L dependent overstimulation, retrograde signaling via GITR-L reduced expression of TLR4 on DCs, thus changing the DC configuration to be less sensitive for activation by environmental cues [426]. In contrast to murine studies, GITR ligation on human T_reg_ cells did not abrogate their suppressive function [427]. Besides T_reg_ cells, stimulation via GITR induced proliferation and cytokine production of effector T cells during priming and within the tumor microenvironment [423,428,429,430,431]. On top, stimulation via GITR was able to break T cell tolerance leading to autoimmune conditions [432,433]. However, adjusted dosing and antigen context may enable the use of the GITR:GITR-L axis as an interesting target to boost the cytotoxic CD8^+^ T cell to T_reg_ cell ratio and break self-tolerance against tumors [434].

Another important signaling axis at the DC:T cell interface is OX40L:OX40 [435,436,437]. Expression of OX40L (CD134-L; CD252) has been described on APCs including DCs, B cells, and macrophages, but also on multiple other activated cell types [435,438,439]. On DCs in particular, OX40L was upregulated following engagement of CD40 [435,437]. On the other hand, OX40 (CD134) expression was detected on T cells following TCR engagement and on T_reg_ cells [435,437,440,441]. Although multiple studies described a distinct role for the OX40L:OX40 interaction during the priming of IL-4 producing Th2 cells, other studies demonstrated that lack or binding competition within the OX40L:OX40 axis is detrimental for both, Th1 and Th2 responses [437,442,443,444,445,446,447]. In general, OX40 is thought to promote CD4^+^ T cell survival [448,449]. While OX40 signaling was important for T_reg_ cell homeostasis and survival, their suppressive capacity was reduced [440,441,450,451,452,453]. With respect to cytotoxic CD8^+^ T cells, simultaneous application of agonistic αOX40 or αCD27 antibodies together with αPD-L1 blockade forced exhausted cytotoxic CD8^+^ T cells to overcome quiescence even though this treatment led to contraction of the effector cell pool in the sustained presence of antigen [454]. As reviewed in detail elsewhere, this dual role recommends the OX40L:OX40 axis as an interesting target for therapeutic intervention during both, autoimmune conditions and cancer [436,455,456]. In summary, OX40:OX40L may constitute a context dependent molecular switch allowing for avoidance of detrimental Th2 responses fueling tumor-supporting chronic inflammation [457].

Although many of the receptors on the T cell side are considered as activators, their role is not completely redundant. Utilizing a model of influenza infection, an early study indicated that CD27 and to a lesser extend 4-1BB (CD137) are essential during initial priming of virus-specific cytotoxic CD8^+^ T cells, whereas the formation of memory was dependent on CD27, 4-1BB, and OX40 [458]. Moreover, in parallel to CD27, stimulation via 4-1BB and OX-40 during priming dictates the capacity of cytotoxic CD8^+^ T cells for secondary expansion during recall responses [458].

T cells can also regulate DC activity. The prime example for regulatory surface molecules is the CD40:CD40L axis. CD40 is present on multiple cell types including DCs, macrophages, T cells, and B cells, non-immune cells, but also some cancer cell types, where CD40 ligation led to growth inhibition [343,459,460]. The importance of CD40 (alias TNFRSF5) ligation on DCs by CD40L (CD154) expressed on helper CD4^+^ T cells during the induction of productive cytotoxic CD8^+^ T cell responses was early demonstrated by multiple studies [343,346,349,461]. Although CD4^+^ T cell help was dispensable for primary expansion and differentiation of cytotoxic CD8^+^ T cells, lack of CD4^+^ T cell help during cytotoxic CD8^+^ T cell priming abolished the secondary re-call capacity of T cells after antigen encounter in the periphery [330,332,344,462]. Ligation of CD40 on DCs by CD40L on CD4^+^ T cells licenses DCs for productive cytotoxic CD8^+^ T cell priming. Licensed DCs translate the received help signal via efficient IL-12 secretion and surface CD70 to subsequently activate and differentiate cytotoxic CD8^+^ T cells, while imprinting their recall capacity [463]. As it has recently been resolved and as we have discussed in Section 4.1, XCR1^+^ cDC1 serve as the central mediator allowing for assembly of the Th cell:DC:CTL platform. Interestingly, while priming of cytotoxic CD8^+^ T cells without CD40 activation rather drove tolerogenic responses, parallel delivery of a minimal CD8 epitope peptide and an agonistic CD40 antibody induced strong cytotoxic CD8^+^ T cell responses [464]. On the other hand, CD40 stimulation without the parallel inclusion of a tumor antigen vaccination promoted the deletion of tumor specific cytotoxic CD8^+^ T cells [465]. Thus, ligation of CD40 represents a powerful but context-dependent tool for breaking tolerance against tumor antigens. Interestingly, a recent study showed that cytotoxic CD8^+^ T cell responses primed by licensed DCs only required activating cytokines, such as IL-12 and IL-18, to induce cytotoxic CD8^+^ T cell intrinsic effector molecule production, including Granzyme B and IFNγ, without the need of MHC-I restricted antigens during recall responses [466]. Thus, it is likely that functional cytotoxic CD8^+^ T cell determinants are epigenetically imprinted during priming by licensed DCs. Additionally, a direct role of CD40 ligation on cytotoxic CD8^+^ T cells was described for cytotoxic CD8^+^ T cell memory formation [459]. Nevertheless, manipulation of stimulatory or inhibitory pathways might bear the danger of autoimmune conditions. In the case of αCD40 antibodies, the most frequent side effect is the development of a cytokine release syndrome causing fever or rigors shortly following systemic application [467]. The use of agonistic CD40 antibodies in clinical trials, their mode-of-action and safety profiles were discussed by Vonderheide and Glennie [467].

Another powerful pair of molecules acting downstream of the CD40:CD40L axis in the DC:T cell communication is CD70:CD27, since mice showing constitutive expression of CD70 on CD11c^+^ cells induced robust cytotoxic CD8^+^ T cell responses leading to tumor eradication without additional application of adjuvants [468]. While CD27 is already expressed on naïve T cells, CD70 is upregulated on DCs following TLR or CD40 stimulation [469,470]. In general, CD70 is utilized by DCs to transmit signals received via CD40 ligation by CD40-L expressed on helper CD4^+^ T cells to cytotoxic CD8^+^ T cells [471,472]. There, CD27 was demonstrated to be essential for the generation of effector and memory cytotoxic CD8^+^ T cells following initial priming even in the absence of IL-12 and type I IFNs [473,474]. Interestingly, interaction of CD70 with CD27 downregulated CD70 on DCs, thereby providing a negative feedback-loop potentially preventing T cell overactivation [475]. With respect to CD4^+^ T cells, CD70 on murine cDC1 was sufficient for Th1 polarization or memory formation of helper CD4^+^ T cells even in the absence of IL-12, the Th1 polarization master cytokine, and type I IFNs [469,476,477]. Furthermore, one study indicated that antigen presentation by cDC2 only induced IFNγ producing Th1 cells in the presence of cDC1 producing IL-12 [477]. While CD70:CD27 co-stimulation was described to counteract naïve CD4^+^ T cell to T_reg_ cell conversion, continuous stimulation fostered clonal, IL-2 driven T_reg_ cell expansion, T cell exhaustion and depletion [478,479,480]. Thus, beneficial effects following manipulation of CD70 expression on DCs by adjuvant application or direct stimulation of CD27 utilizing monoclonal, agonistic antibodies may be duration- and dose-dependent. Nevertheless, the use of CD27 agonists synergized with PD-1 blockade, by boosting cytotoxic CD8^+^ T cell-based anti-tumor immunity emphasizing the potential of combinatorial agonistic and antagonistic therapeutical approaches [481]. The complex biology of CD70:CD27 interaction is reviewed nicely elsewhere [479].

While most of the aforementioned checkpoints exert their major influence during T cell priming, other regulatory axes have been identified primarily regulating T cell activity directly within the tumor tissue including VISTA, PD-1, and the PD-1 ligands PD-L1 and PD-L2.

Programmed cell death protein 1 (PD-1; CD279) is expressed on various cell types including B cells, NK cells, some myeloid cells, and T cells after antigen recognition via the TCR, but not on naïve T cells [482,483,484]. In general, PD-1 directly counteracts TCR signaling, but affects mainly CD28 signaling via recruitment of the phosphatase SHP2 [485,486,487]. In the case of rapid antigen clearance, PD-1 levels are dynamically downregulated [488,489]. However, sustained antigen exposure as observed during cancer maintains PD-1 expression [488,489,490]. Thereby, antigen dose and PD-1 concomitantly contribute to the decision between T cell exhaustion and memory [484,488]. On exhausted cytotoxic CD8^+^ T cells, PD-1 exhibited high and sustained expression [488]. However, PD-1 blockade resulted in the rescue of exhausted cytotoxic CD8^+^ T cells in a CD28-dependent fashion and increased the numbers of CD28^+^ T cells [485,488,491]. Hence, CD28 may serve as a biomarker for the efficacy of the PD-1 blockade [491]. Further, metabolic downstream effects of PD-1 signaling may act in synergy with the tumor microenvironment. While stimulation via the TCR and CD28 efficiently switches the T cell metabolism to glycolysis, PD-1 signaling counteracts glycolysis and supports fatty acid metabolism in helper CD4^+^ T cells, thereby reducing the energy available for T cell activity [486,492,493,494]. This feature of PD-1 may be of particular importance within the tumor microenvironment, which is highly competitive concerning available energy sources [492]. Thus, besides direct PD-1 blockade, manipulation of downstream metabolic pathways may promote T cell function [492,495].

PD-1 has two ligands: Programmed cell death protein 1 ligand 1 (PD-L1; alias CD274; B7-H1) and PD-L2 (alias B7-DC; CD273) [496,497,498,499]. Whereas PD-L1 is broadly expressed on T cells, B cells, DCs, macrophages, and non-hematopoietic cells, PD-L2 expression is mainly restricted to APCs including DCs, macrophages, and B cells [483,484,496,497,498,499]. PD-L1 and PD-L2 were recognized to be expressed by tumors very early, thereby equipping tumors with a tool to silence T cell immunity [498,499]. In particular, manipulation of the PD-1:PD-L1 axis by PD-L1 blockade or PD-L1 overexpression substantially inhibited or promoted tumor development in mice, respectively [500]. Further, a study by the group of Sharpe indicated that the ability and requirement for PD-L1 expression by tumor and non-tumor cells differed between different tumor types to mediate immune escape and inhibit cytotoxic CD8^+^ T cell responses [501]. The PD-1:PD-L1 axis may also be of particular importance for re-silencing of initially hot tumors, since the expression of PD-L1 was found to be sensitive for the cytokines IFNα, β, and γ as well as IL-12, which require the presence of different activated effector cells [502]. Since IL-12 was not able to upregulate PD-L1 in IFNγ-deficient mice, IFNγ may be the main driver for PD-L1 upregulation. As we will discuss later, DC-derived type I IFNs and IL-12 are major activators of NK cells, cytotoxic CD8^+^ T cells and Th1 cells, thereby promoting the production of the intracellular defense hallmark cytokine IFNγ [503]. While under normal circumstances PD-L1 may contribute to resolution of inflammation, this regulatory circuit can be hijacked by tumor cells to escape NK cell, cytotoxic CD8^+^ T cell, and Th1 cell surveillance. Further, tumor-derived PD-L1 fostered T cell apoptosis [504]. Since the PD-1:PD-L1 axis exerts multiple functions within the tumor environment, combinatorial therapeutic approaches allowing for simultaneous manipulation of T cell priming in secondary lymphoid organs and modulation of effector functions in the highly suppressive tumor microenvironment were therefore suggested early to improve cancer therapy. In particular, CTLA-4 and PD-1 inhibit T cell activity via non-redundant mechanisms [486]. Thus, following clinical studies illustrating the promising potential of checkpoint inhibitor monotherapies manipulating the PD-1:PD-L1 axis, dual manipulation of non-redundant pathways was thought to be an attractive treatment option [505,506,507]. Simultaneous blockade of CTLA-4 and PD-1 for the treatment of patients suffering from metastatic melanoma improved efficacy, but additionally caused higher rates of adverse effects compared to checkpoint inhibitor monotherapy [508]. Besides PD-1, PD-L1 also interacts with CD80, thereby blocking T cell proliferation [509].

Besides direct turn-down of cytotoxic CD8^+^ T cell and helper CD4^+^ T cell effector functions, PD-L1 is an important regulator of different aspects of induced T_reg_ cells including initial conversion by APCs, T_reg_ cell maintenance and suppressive capacity [510]. There, blockade of PD-1 reversed suppression of melanoma-specific cytotoxic CD8^+^ T cells by T_reg_ cells in human patients [511]. Furthermore, PD-1 constitutes an important regulator of NK cell activity [512]. Thus, releasing the brakes of NK cells in tumor variants characterized by downregulated or absent MHC-I by PD-1 pathway manipulation might counteract immune evasion caused by tumor-blinded cytotoxic CD8^+^ T cells [513].

With respect to DCs, retrograde signaling via PD-L1 and PD-L2 induced a regulatory phenotype in bone marrow-derived DCs characterized by increased IL-10 production and counteracted maturation [514]. Thus, blockade of PD-L1 or PD-L2 might also reprogram the DC compartment directly, thereby boosting T cell immunity. Similarly, engagement of PD-L1 expressed on T cells led to anergic, IFNγ negative cytotoxic CD8^+^ T cells, and helper CD4^+^ T cells with reduced Th1 and increased Th17 differentiation. These cells finally induced an alternative activated (‘M2’) macrophage program thwarting anti-tumor immunity [515]. Since the number of clinical studies and the proposed modes of action are very complex, we would like to refer to other recent reviews for further reading [484,513].

V-domain Immunoglobulin Suppressor of T cell activation (VISTA; alias PD-1H, Gi24, DD1α, Vsir, or Dies1) is an inhibitory molecule expressed on many cell types including DCs, other myeloid cells, CD4^+^ and CD8^+^ T cells as well as tumor cells [516,517,518]. VISTA acts as both, receptor and ligand [519]. In humans, VISTA is an efficient suppressor of human T cell activation and additionally induces FoxP3, thus contributing to T_reg_ cell conversion [516]. Since VISTA levels are often increased within the tumor microenvironment, VISTA blockade may act comparable to PD-1 or PD-L1 primarily within the tumor although in a non-redundant manner [516,520]. There, VISTA activity potently blocks T cell function [518,521]. Conversely, VISTA-deficient mice were prone to develop autoimmune conditions, exhibited APCs with an increased inflammatory capacity, a highly activated T cell compartment increasing over time, and rapid pro-inflammatory cytokine production following re-activation [522,523,524].

During tumor therapy, blockade of VISTA boosted recruitment of tumor-specific T cells and activated DCs in the tumor microenvironment, while decreasing the number and activity of myeloid derived suppressor cells and tumor-specific T_reg_ cells [525,526]. Further, VISTA was upregulated by p53 activity contributing to the clearance of dead cells by boosting phagocyte functions [518]. Thus, VISTA might be important on different cell types including apoptotic cells, phagocytes and T cells, to retain unresponsiveness to antigens derived from dead cells and consequently for the immunological silencing [517,521]. However, the very same mechanisms might be hijacked by cancer cells to escape immune surveillance [517,521]. Thus, VISTA blockade in the tumor microenvironment may change the anti-inflammatory context during uptake of VISTA^+^ tumor cells and thereby impeding the subsequent suppression of T cell immune responses induced by VISTA^+^ phagocytes. Finally, VISTA and PD-1 pathways were increased following CTLA-4 blockade in patients suffering from prostate cancer indicating that the manipulation of VISTA also acts in synergy and non-redundant to CTLA-4 blockade [527]. In summary, VISTA represents an interesting checkpoint for modulating the immune escape of cancer cells. The complexity of VISTA biology and its potential for therapeutic approaches was nicely summarized in other recent reviews [516,519].

Although this review includes many prominent members orchestrating cross-talk at the molecular interface of DCs and T cells, it can only provide a brief insight into the numerous molecules involved and the complexity of the DC:T cell communication including options to interfere. Thus, the study of detailed reviews on these aspects are suggested for further reading [513,528,529,530].

#### 4.2.3. Signal 3: DC Cytokine Secretion Determines T Cell Polarization

In parallel to pMHC presentation and co-stimulation (or co-inhibition) by surface molecules, DCs are able to secrete a variety of soluble factors mostly cytokines, chemokines, and metabolites [106,531,532]. The specific patterns of secreted soluble mediators direct subsequent T cell polarization.

Murine cDC1 secrete major amounts of IL-12, thereby fostering cytotoxic CD8^+^ T cell and helper CD4^+^ Th1 cell differentiation and the formation of their specialized effector modules, which are summarized in Figure 1 [59,60,533,534,535,536,537,538,539,540,541,542,543]. Furthermore, cDC1 induce T_reg_ cells following secretion of tumor growth factor β (TGFβ) [63,531,544]. In particular, one study highlighted the importance of a BTLA^+^ cDC1 to convert T cells to peripheral T_reg_ cells, while BTLA^-^ cDCs lacked this functional feature even under steady state conditions [40]. Further, the production ability of IL-12 and IFNα by BTLA^+^ cDC1 shifted to IL-4 and TGF-β during tuberculosis infection, thereby favoring Th2 and T_reg_ cell polarization [545]. Additionally, early studies demonstrated that antigen presentation of DEC205^+^ cDC1 without inflammatory stimuli resulted in T cell tolerance [55,56,62,63,544]. cDC1 also maintain cross-tolerance of cytotoxic CD8^+^ T cells by either inducing anergy or clonal deletion of self-reactive T cells [546,547,548].

While cDC1 are considered as major contributors to the defense against intracellular threads, including viral and malignant diseases, cDC2 are important for the polarization of Th2 and Th17 T cells, thereby acting as major regulators in the defense against extracellular pathogens [69,542,549,550]. Th17 cells are polarized via TGFβ, IL-21, IL-23, IL-6, and IL-1β, where IL-23 is rather important for Th17 cell expansion, maintenance, and effector functions than the initial polarization [551,552,553,554,555,556,557,558]. Since TGFβ is needed for both, T_reg_ and Th17 cell polarization, IL-6 was identified as the molecular switch suppressing T_reg_ cells and fostering Th17 fate [1,551,559]. Hence, IL-6 and TGFβ can be considered as the master regulators of Th17 cell development. Furthermore, IFNγ and IL-4 act as TGFβ antagonists, thereby providing the basis for Th1 and Th2 polarization [553]. Interestingly, multiple studies suggest functionally distinct populations within the murine cDC2 subset. While murine Notch2-dependent cDC2 were found to produce IL-23, thereby fostering ILC3, Th17 cells, and follicular T helper cells (Tfh) promoting humoral immune responses, KLF4-dependent cDC2 were identified as instructors of Th2 cell responses [25,66,68,70,560]. Of note, the DC mode of action during Th2 priming is under debate. Since DCs have not been described to express the Th2 master cytokine IL-4, it is speculated that cDC2 either contribute to Th2 differentiation via absence of Th1/Th17 cytokines or by an unknown signal [561,562]. Alternatively, IL-4 could be provided by differentiating T cells in an autocrine manner or by bystander cells [561,562]. cDC2 also contribute to the maintenance of tolerance. While peripheral cDC2 were described to expand already primed T_reg_ cells, cDC2 were suggested to re-circulate to the thymus, thereby acting on central CD4^+^ T cell tolerance [63,563].

In contrast to mice, human cDC2 are also described to efficiently cross-prime cytotoxic CD8^+^ T cell responses and produce IL-12, although the capacity of cDC2 exerting these functions compared to cDC1 is a matter of debate [87,371,564,565,566,567,568,569,570,571,572]. However, a comprehensive summary of cytokines needed for and produced by polarized T cells is depicted in Figure 1. Please note, that this figure does only include hallmark cytokines and therefore does not claim completeness. Cytokine production is usually not unique for one cell type, but rather occurs in some cell types at higher rates leading to their association with a specific cytokine [371]. Furthermore, the outcome in studies assessing cytokine production is critically dependent on the investigated micromilieu/organ as well as the applied stimuli and time point of measurements [45,573,574].

Cytokine secretion of DCs affects also other cell types than T cells. For example, MyD88-deficient DCs unable to trigger IL-12 production were found to tremendously affect the IFNγ response by NK cells and this adverse effect could be reversed via simple IL-12 treatment [575]. Further, reciprocal effects of NK cells influencing DCs have been described. In a study by Böttcher et al., NK cells were important for recruiting cDC1 into the tumor tissue in a CCL5- and XCL1-dependent manner, thereby promoting tumor control [576]. While NK cells are the innate variant of CD8^+^ cytotoxic T cells, other innate lymphoid cells (ILCs) have been identified, which resemble their cognate T cell subset with respect to transcription factor requirement and cytokine production [577,578]. Hence, cDC1 support ILC1 via IL-12 (counterpart of Th1), whereas cDC2 secrete IL-23 boosting ILC3 and Th17 cells [577]. ILC2 secrete IL-13 to foster Th2 and cDC2 driven effector modules [577].

In addition to polarizing cytokines, DCs are able to secrete stimulatory or inhibitory cytokines, such as type I interferons (IFN-I) or IL-10, respectively [106,579,580,581,582]. Since the interferon regulatory factor 8 (IRF8), which is critical for the development of cDC1, boosted the IFN response as a consequence to sensed IFN, cDC1 are ideally equipped for both, IFN production and responsiveness [583]. Type I IFNs were critical for cross-presentation to cytotoxic CD8^+^ T cells and enhanced maturation, antigen retention, uptake of apoptotic material, cDC recruitment, survival and DC induced rejection of tumors [584,585,586,587,588,589,590]. Furthermore, this is due to an autocrine type I IFN signaling [584,591]. Thus, induction of type I IFNs serves as ideal target for adjuvant therapy. However, experiments utilizing bone marrow derieved DCs demonstrated that sustained type I IFN exposure induced PD-L1 expression [592]. In addition to type I IFNs, murine and human cDC1 seem to be specialized for IFNλ (IL-28/IL-29; type III IFN) production [573]. In particular, human cDC1 responded by IFNλ2 (IL-28A) and IFNλ1 (IL-29) secretion after stimulation with poly(I:C) [573]. A recent study indicated that cDC1-dependent IFNλ signatures, in particular of IFNλ1 (IL-29) and its receptor, were associated with superior disease outcomes in breast cancer [593]. This might be explainable by the capacity of IFNλ to drive a Th1 environment via boosting production of IL-12, IFNγ and cytotoxic CD8^+^ T cell-recruiting cytokines [593]. On top, this study showed that TLR3 ligation allows for kick-starting IFNλ responses by tumor-associated cDC1 [593]. Finally, murine cDC1 or cDC2 were shown to mount cytotoxic CD8^+^ T cell responses via IL-27 following stimulation with poly(I:C) (TLR3 ligand) or R848 (TLR7 ligand), respectively [162].

Besides cytokines and chemokines, DCs secrete or manipulate other soluble factors, thereby influencing their environment. For instance, expression of the aldehyde dehydrogenase (Aldh) allows for the conversion of vitamin A into retinoic acid, which was tolerogenic in multiple studies and enabled the induction of T_reg_ cells [531,532,594,595]. Beyond, DCs can also manipulate the availability of the essential amino acid tryptophan by metabolizing tryptophan to kynurenine via indoelamine-2,3-dioxygenase (IDO) enzyme activity, which is in particular found in murine cDC1 [596,597,598]. However, IDO activity can be induced via TGFβ in both, cDC1 and cDC2 [599]. Additionally, inflammatory signals, such as the cytokines IFNλ, IFNγ, IFNβ, and TNFα, were all described to upregulate IDO [600,601,602,603,604]. In general, it is believed that tolerogenic effects downstream of IDO activity are mediated via the suppression of effector T cells and hyperactivation of T_reg_ cells by tryptophan starvation and by the resulting enzymatic products [554,603,605,606,607]. Furthermore, T_reg_ cells regulated tryptophan metabolism in DCs in a CTLA-4-dependent fashion, thereby potentially creating a DC:T_reg_ feedback loop [596,606]. Thus, application of IDO inhibitors, such as 1-methyl tryptophan, may be useful to enhance the stimulatory capacity of cDC1 in particular. A schematic and simplified summary of key activating, inhibitory and polarizing soluble factors is depicted in Figure 1.

## 5. DCs in the Tumor Microenvironment: Tipping the Scales during Anti-Cancer Immune Responses?

The tumor microenvironment (TME) is a heterogeneous landscape integrating the dynamic interplay of tumor intrinsic defense mechanisms, tissue-dependent factors, the microbiome and various cell types besides cancer cells including different immune cells, fibroblasts, endothelial cells and neurons [608,609,610,611,612]. Multiple immune cell types with immuno-suppressive functions have been identified within the tumor-infiltrating cell pool including monocytes, tumor-associated macrophages (TAMs), pDCs and T_reg_ cells, but also cDCs [89,609,613,614]. However, several studies indicate that specific immune signatures are key to inhibit or even stop disease progression. For instance, infiltration of cytotoxic CD8^+^ T cells into primary human tumors is associated with beneficial disease outcomes [611,612,615]. With respect to DCs, cDC1 constitute a scarce cell population within tumors [613]. However, the presence of their transcriptomic signature and an overall higher abundance within tumor lesions are associated with a better disease outcome [616]. Further, CD103^+^ or CD141^+^ DCs in mice or humans are critical to transport tumor cargo to draining lymph nodes in a CC chemokine receptor 7 (CCR7) dependent manner [616]. Additionally, multiple studies indicated that cDC1 are crucial to mount productive anti-tumor immune responses including responses following therapeutic checkpoint inhibitor or chemotherapy treatment regimens [39,576,613,617,618,619,620]. Thus, cDC1 and their transcripts might serve as prognostic biomarker for cancer therapy. Additionally, a recent study by the group of Krummel identified cDC2s in mice and men trafficking from tumors to draining lymph nodes presenting tumor antigens to conventional CD4^+^ T cells [621]. However, these cDC2s failed to unleash productive anti-tumor CD4^+^ T cells, but their functionality was restored following the depletion of T_reg_ cells [621]. Thus, the abundance of cDC2 in relation to T_reg_ cells was predictive for disease outcome [621]. This importance of cDCs for cancer immuno-surveillance is highlighted by the finding that specific temporal ablation of all cDCs indicated that cDCs are in charge to counteract metastasis, since cDC-depleted mice were more susceptible for the formation of metastatic lesions in a murine lung colonization model [622]. Hence, cDCs might survey and impede the formation of (pre-) metastatic niches.

cDC1 can recruit CTLs and NK cells to the tumor site and vice versa [576,613,619,623]. While migratory cDCs allow for the shuttling of tumor cargo, which was potentially acquired via the specialized cDC1 receptor for necrotic material CLEC9A, to the tumor-draining lymph nodes facilitating initial T cell priming, tumor-located cDC1 enable the reactivation of cytotoxic CD8^+^ T cells within the tumor [41,613,617,622]. Thus, simply paucity in tumor-located and migrating cDCs and effector cells might promote disease progression. This shortage can be caused by tumors via direct exclusion of effector cells and/or by manipulating e.g., the CCR7-dependent lymphoid organ homing of migratory DCs [7,624,625,626]. Combined treatment with Fms-like tyrosine kinase 3 ligand (FLT3-L) and the TLR-ligand poly(I:C) expanded and simultaneously activated the cDC pool, thereby overturning cDC paucity at tumor sites [617].

However, a recent study by the group of Merad demonstrated that not only the quantity of tumor-located DCs, but also their quality is important [89]. By using single-cell RNA sequencing of human and murine non-small cell lung cancers, they identified DC signatures enriched in maturation-associated (CD40, CCR7 and IL-12b) and immunoregulatory molecules (PD-L1, PD-L2 and CD200) summarized under the term mregDCs. This transcriptional program developed in cDC1 and cDC2 following acquisition of tumor antigens [89]. Further, upregulation of PD-L1 was dependent on AXL in mregDC1s. Besides, the TLR sensor portfolio was strongly downregulated in mregDCs compared to canonical cDC1 and cDC2 in humans and mice, potentially rendering these cells rather inert to subsequent TLR stimuli [89]. Finally, this study provides evidence that IL-12 production in mregDC1s is tightly controlled either positive or negative by IFNγ or IL-4, which are hallmark cytokines for either cytotoxic CD8^+^ T cell, helper CD4^+^ Th1, or NK cell-driven intracellular defense modules or helper CD4^+^ Th2 cells, respectively [89]. Thus, IL-4 neutralization represents an interesting tool for favoring IL-12-producing cDC1-driven anti-tumor effector modules facilitating the expansion of functionally active tumor infiltrating T cells, thereby limiting tumor progression [89,457]. In accordance with these results, a recent study utilizing single cell sequencing validated that tumor located cDC1, cDC2 and migratory DCs possess a highly activated phenotype including the expression of 4-1BBL, OX40-L, IL-1β, and TNFα compared to their respective lymph node clusters [614]. Similarly, this T cell stimulatory phenotype was accompanied by the expression of immuno-regulatory molecules. While this remodeling did not extend to the DC subsets located in the lymph nodes, the regulatory phenotype of the tumor myeloid compartment increased over time [614]. This argues that DCs are initially activated within the tumor microenvironment of select tumor entities, but their stimulatory properties are overruled over time potentially leading to loss of tumor surveillance [614].

## 6. Harnessing the Potential of the Immune Systems’ Generals during Immunotherapeutic Approaches

Since DCs have been recognized as the principle cell type able to trigger responses of naïve, antigen-specific T cells, great efforts have been made to harness DCs for cancer immunotherapy [3,106,109,627,628,629,630].

### 6.1. Autologous DC Transfer Approaches for the Treatment of Malignancies

A fundamental breakthrough was the discovery that monocytes can be differentiated in the presence of granulocyte-macrophage colony stimulating factor (GM-CSF) and IL-4 ex vivo to moDCs at high numbers making DCs accessible for immunotherapeutic approaches [17,18,631,632,633]. To allow for the induction of immune responses, maturation of moDCs is a prerequisite. This can be achieved by different strategies including the treatment of moDCs with agonistic αCD40 antibodies, the application of TLR ligands or via usage of the classical moDCs maturation cocktail including IL-1β, IL-6, TNFα, and Prostaglandin E2 (PGE_2_) [109,631,634,635,636,637,638]. Before adoptive transfer, moDCs can be loaded with antigen(s) of choice. Therefore, several techniques have been developed including MHC peptide pulsing, co-incubation with soluble proteins or whole tumor cell lysates or the transfection with tumor DNA, whole tumor or epitope-specific mRNAs [109,631,639,640,641,642,643,644,645,646,647]. Transfection methods, including the current gold standard of mRNA electroporation, additionally allow for the introduction of mRNA coding for functional proteins, thereby enabling the creation of ‘designer moDCs’ with an optimized phenotype [631,643]. For instance, co-transfection of antigen and a constitutively active form of the NF-κB pathway activator IKKβ increased the inflammatory potential of moDCs [648,649,650]. Designer moDCs produced elevated amounts of cytokines including IL-12, thereby boosting NK and cytotoxic CD8^+^ T cell crosstalk, exhibited upregulated expression of activation markers and co-stimulatory molecules and finally induced cytotoxic CD8^+^ T cells with a higher lytic capacity [648,649,650]. Thus, designer moDCs represent an interesting tool for further enhancing the clinical efficacy of moDC-based vaccines. To date, moDC monotherapies have delivered promising clinical results in the treatment of cancer, while exhibiting only moderate adverse effects rendering combinatorial approaches including DC vaccination and checkpoint blockade very interesting, in particular for the treatment of patients with a high tumor burden [631].

So far, moDCs have been employed in the clinic to treat a variety of different tumor entities including mesothelioma, prostate cancer, non-small cell lung cancer, hepatocellular carcinoma, acute myeloid leukemia, Her2-expressing breast cancer, colon and colorectal cancer, renal carcinoma, pediatric brain cancer and neuroblastoma, glioblastoma, unresectable pancreatic cancer, B cell lymphoma and most commonly late stage melanoma [647,651,652,653,654,655,656,657,658,659,660,661,662,663,664,665,666]. In general, response rates ranging from ~7% to ~32% have been described by different studies [631]. Owing to different pre-treatments, culture conditions, used antigenic material, various tumor entities, treatment schedule and route of administration, efficacy cross-comparisons of clinical trials are difficult. However, an early review from 2009 summarized the results for treating malignant melanoma with autologous DC preparations [667]. Across all evaluated studies, 3% of patients exhibited complete responses, 6% partial responses, while 21% displayed stable disease, thus adding to a clinical response rate of 30% [667]. While this analysis did not emphasize a specific route of administration, the stable disease phenotype was significantly associated with the induction of antigen specific T cells [667]. DCs have the power to initiate de novo T cell responses and checkpoint inhibition has been described to boost the functionality present T cell responses [668,669,670]. This raises the hope that the therapy responders during DC- and checkpoint inhibitor regimens are in principle not the same group of patients. Thus, combinatorial approaches might allow for high response rates across patients. Since autologous DC vaccines are in general considered safe, they are an attractive fit to team up with checkpoint inhibition, which can be associated with severe side effects as we have discussed in Section 4.2.2 [631]. The general working principles, ongoing and published clinical trials, their outcome and overall safety profiles were recently nicely summarized by Dörrie et al. [631].

Besides monocytes, CD34^+^ stem cells can be employed as source for DC differentiation. Since CD34^+^ cells can be mobilized from the bone marrow into the peripheral blood before leukapheresis via a simple granulocyte colony-stimulating-factor (G-CSF) treatment, CD34^+^ cells represent an interesting source for the massive generation of ex vivo-derived DCs [671,672,673]. Following cultivation in the presence of GM-CSF and TNFα, a heterogeneous DC mixture arises including cells with a Langerhans cell phenotype (CD1a, E cadherin and Birbeck granules) and a DC population derived from CD14^+^ cells [674,675]. CD34^+^ cell-derived DC vaccines displayed also promising results in inducing antigen specific T cell responses [676,677,678,679,680]. Usage of autologous CD34^+^ stem cell based vaccines has already been employed in the clinic for the treatment of metastatic melanoma allowing for the induction of melanoma-antigen specific T cell responses correlating with an increased survival [666,672,676,681,682].

Additionally, recent studies providing a sophisticated cell culture protocol or engineered niches enable the differentiation of all three major DC subsets from CD34^+^ cells including cDC1, cDC2, and pDCs [683,684]. Administration of FLT3-L expanded primary DCs from the precursor pool of healthy volunteers and cancer patients and recruited immediate DC precursors but no earlier differentiation stages to the circulation [685,686]. Finally, FLT3-L also expanded monocytes in the peripheral blood [686]. Thus, FLT3-L might be a perfect fit for both, future moDC or primary DC, treatment regimens. Recombinant human FLT3-L has also been tested in a clinical trial in combination with a DC targeting DEC205:NY-ESO-1 fusion in patients following melanoma resection as a preventive treatment to abolish disease recurrence [687]. In contrast to DEC205:NY-ESO-1 monotherapy, additional application of FLT3-L led to a 15 to 200 fold expansion of the myeloid cell pool measured in the blood, higher αNY-ESO-1 antibody titers, with both treatments were well tolerated [687].

The potential of primary DC populations in the course of autologous transfer approaches for cancer immunotherapy has also been exploited. In 2012, a pioneer study by the group of de Vries employed pDCs purified from peripheral blood via BDCA-4 for autologous transfer following tumor peptide loading for the treatment of patients suffering from metastatic melanoma [76]. In summary, this study demonstrated that pDCs possessed functional lymphoid tissue migration, induced an IFN signature and induced anti-vaccine CD4^+^ and CD8^+^ T cell responses, while exhibiting only minimal adverse effects [76]. As we have discussed in Section 2, it remains elusive if potentially included pre-cDCs (AS DCs) in the cell preparation represented the therapeutically active fraction during therapy. However, validation of this hypothesis would highlight that such treatment regimens are applicable using minimal amounts of cells, rather than challenging the potential of autologous primary DCs approaches. Current clinical trials have also assessed the potential and safety of autologous cDC2 transfer approaches for the treatment of metastatic prostate cancer and melanoma [688,689]. While both vaccines were safe not inducing considerable adverse effects, 28% of melanoma patients exhibited a long-term progression free survival, which coincided with the development of potent cytotoxic CD8^+^ T cell responses.

The use of moDCs, CD34^+^-derived DCs and primary DCs for autologous transfer approaches for the treatment of cancer therapy was recently summarized elsewhere [109,627,631,643,690,691]. In contrast to labor-intensive autologous cell transfer approaches, direct targeting of DCs in vivo is not regularly employed in the clinics, even though this could allow for harnessing specialized DC subsets in their natural environment.

### 6.2. Antigen Targeting Enables the Induction of Antigen Specific T Cell Effector Modules Following Antigen Delivery to DCs In Vivo

Although many different strategies have been developed to introduce tumor-derived antigens into the immune system, most of these approaches do not allow for specifically addressing antigens directly to DCs. The early discovery of the monoclonal antibody clones 33D1 and NLDC-145 directed against the endocytic CLRs DCIR2 and DEC205 expressed on murine cDC2 or cDC1, respectively, fostered the idea to develop antibody-based shuttle systems allowing for direct delivery of antigens of choice to distinct DC populations in vivo [55,62,692,693]. Thereby, the DC subset intrinsic capability for the orchestration of specific T cell effector modules can be exploited. Since a recent review of our group already thoroughly summarized the plethora of antigen targeting studies performed in the last decades (see Lehmann et al., Vaccines, 2016), this review will focus on suitable receptors, DC subset specific expression patterns, different antibody-based delivery systems and the rationale for vaccine design [109].

#### 6.2.1. The Principles of Antigen Delivery to DCs Utilizing Antigen-Coupled Targeting Antibodies In Vivo

**Figure 2:**
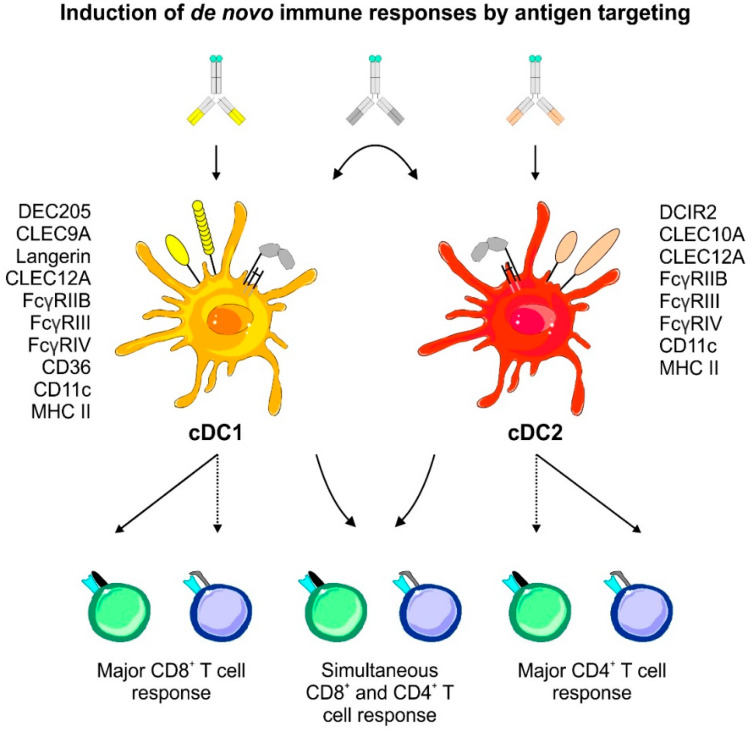
Delivery of antigens by targeting antibodies directed against select endocytic receptors expressed on DC subpopulations induces defined T cell responses in mice in vivo. Coupling of antigens to monoclonal targeting antibodies specifically recognizing endocytic dendritic cell (DC) surface receptors enable the loading of select DC subpopulations with an antigen of choice following in vivo application. While targeting antigens to murine cross-presenting cDC1 via DEC205, CLEC9A, Langerin (CD207), and CD36 were described to result in major CD8+ T cell responses following peptide:MHC-I presentation, antigen delivery via DCIR2 and CLEC10A expressed on cDC2 induce a strong CD4+ T cell response. Moreover, simultaneous delivery of antigens to cDC1 and cDC2 via FcγRIIB, FcγRIII, FcγRIV, CD11c, and MHC-II allow for the induction of concomitant CD8^+^ and CD4^+^ T cell responses. DC = Dendritic cell; cDC = conventional dendritic cell; MHC = major histocompatibility complex; DCIR = Dendritic cell immunoreceptor; CLEC = C-type lectin receptor; CD = cluster of differentiation; FcγR = Fc gamma receptor. The cellular images are provided and adapted from Servier Medical Art (smart.servier.com). The images are licensed under a Creative Commons Attribution 3.0 Unported License (creativecommons.org/licenses/by/3.0/).

Multiple studies have demonstrated that application of antigen-coupled targeting antibodies directed against endocytic receptors expressed by murine or human DCs allows for loading of DCs with antigen and subsequent manipulation of T cell responses including induction of immunity or tolerance in vivo [55,56,61,62,63,136,544,694,695,696,697]. With respect to cancer immunotherapy, the potential of antigen targeting to different endocytic receptors, including DEC205, DCIR2, CLEC9A, CD36, LOX-1, CD11c, and MHC-II was proven in several murine tumor models [133,137,138,694,695,698,699,700,701,702,703,704,705,706]. In mice and men, most candidate receptors identified belong to the CLR family and we have discussed their major characteristics and expression patterns in Section 3.2 and Table 1. Since cDC1 and cDC2 display discrete CLR expression patterns, selective loading of cDC1 or cDC2 with antigens was demonstrated to induce strong cytotoxic CD8^+^ T cell or helper CD4^+^ T cell responses, respectively [55,56,136,137]. Although the induction of tumor-specific cytotoxic CD8^+^ T cells by cross-presenting cDC1 is assumed pivotal for effective cancer immunity, a study by Neubert et al. showed that targeting of cDC2 was also protective and induced therapeutic immune responses in a model of murine melanoma [137]. Hence, identification and functional testing of target receptors simultaneously expressed on cDC1 and cDC2 may further boost anti-tumor immunity. Such an attractive receptor class for antigen delivery approaches was recently identified in mice [56]. There, targeting antigens to FcγRs, in particular FcγRIV, allowed for simultaneous antigen loading of cDC1 and cDC2 accompanied by concomitant induction of strong cytotoxic CD8^+^ T cell and helper CD4^+^ T cell responses proven by epitope-specific restimulation and in vivo-cytotoxicity assays [56]. Even though FcγRs are expressed on various other immune cells, this study additionally provided evidence that the induction of T cell responses following FcγR targeting relies on cDCs, while other FcγR expressing APCs, including monocytes, pDCs, and B cells, were dispensable [56,154]. Translation of the knowledge acquired using murine antigen targeting approaches to the human system will require the identification and functional testing of suitable targeting receptors displaying expression on cDC1, cDC2, or both populations as has been already demonstrated for CLEC9A, CLEC10A, or CLEC12A [45,100,697]. The overall principle of targeting endocytic receptors on specific DC populations and the respective functional outcome is schematically summarized in Figure 2.

#### 6.2.2. Engineering of Antigen-Coupled Targeting Antibodies and Antibody-Coated Transport Vehicles

Following initial production and characterization, first-generation targeting antibodies were engineered on protein level. Since antibodies are normally not generated in the target species or their production relies on foreign expression systems, antibodies may not be well tolerated by the recipient’s immune system [707,708]. To avoid the induction of unwanted immune responses against the targeting antibody, species matching is of major importance. While the constant antibody regions, including the fragment crystallizable (Fc), can be species-matched by simple cloning procedures, humanization of variable antibody regions requires the use of genetically modified mice or grafting of complementary determining regions into human framework regions [709,710,711]. In transgenic mice, naïve immunoglobulin loci can be replaced by the human immunoglobulin loci, thus allowing humanization of variable regions enabling the generation of a fully human antibody repertoire [712,713,714,715]. Another factor to consider is the potential of IgG targeting antibodies for unspecific interaction with FcγRs. Since the murine IgG1 and human IgG4 isotypes exhibit the lowest binding affinity across the FcγR landscape, generation of recombinant targeting antibodies of the respective isotypes minimizes unspecific binding by FcγRs [716,717]. Further, deglycosylation or single-amino-acid exchanges, for example at Fc glycosylation sites of murine or human (e.g., D265A or N297A) IgG, can modify/abolish FcγR binding and the respective effector functions [717,718,719,720,721]. Thus, it is reasonable to design targeting antibodies with a murine IgG1 or human IgG4 isotype, which are mutated in the Fc glycosylation sites responsible for FcγR binding.

**Figure 3:**
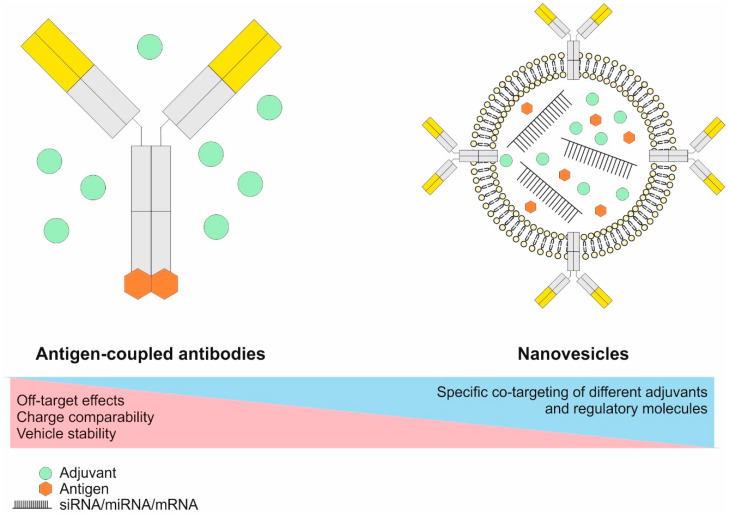
Schematic representation of different targeted carrier-systems. Antibody-based shuttle systems can be employed to specifically address molecules to DCs in vivo. While the antigen can be directly coupled to the targeting antibody, novel approaches might even allow for direct delivery of adjuvants via the shuttle system, thus potentially minimizing unwanted adjuvant off-target effects. Therefore, chemical coupling of adjuvants to the targeting antibody or inclusion of adjuvant and antigens in antibody-coated nanovesicles may provide an elegant solution. Further, the use of nanocarrier-systems might even allow for the delivery of siRNAs and miRNAs to modify the DC function on post-transcriptional level or to directly express proteins of choice following mRNA loading. siRNA = small interfering RNA; miRNA = micro RNA; mRNA = messenger RNA.

Following antibody generation, an antigen of choice can either be included in antibody-coated nanocarriers or directly attached to targeting antibodies via chemical coupling or production of recombinant antibody:antigen fusion constructs (schematically illustrated in Figure 3). In contrast to chemical coupling, recombinant designing allows for control and manipulation of several parameters including antigen dose or location of labelling, thus ensuring comparability of different production lots [109].

Currently, glyco-engineering of antibodies is used to direct and improve antibody effector functions. Although many of these approaches focus on the intrinsic effector modules of the IgG Fc, which are intentionally eliminated in antigen targeting antibodies, glycosylation could influence other aspects including immunogenicity, recognition by sugar-binding receptors, or antibody half-life. The endocytic CLRs expressed on DCs may constitute prime candidates for application of glyco-engineered targeting antibodies. Since glycans, either directly coupled to antigens or labelled to antigen-including nanocarriers, have already been demonstrated to direct antigens to CLRs expressed on APCs, manipulation of targeting antibody glycosylation may further fine tune different aspects of antibody:CLR interaction [722,723,724,725]. These could include binding affinity by parallel recognition of the CLR by the antibody variable region and the sugar moiety by the CLR, binding specificity by minimizing the presence of sugar moieties potentially recognized by other CLRs, intracellular routing during antigen processing or finally induction of signaling by CLRs fostering antigen presentation in an inflammatory context [726,727,728,729,730].

Since antigen presentation by DCs without co-stimulation fosters tolerogenic responses, parallel delivery of adjuvants is considered paramount [56,61,62,63]. Therefore, studies aiming to induce anti-tumor T cell responses relied on the application of targeting antibodies together with adjuvants, such as natural or mimetic TLR agonists. Although it is generally believed that DC activation includes downregulation of the endocytic capacity or expression of select receptors itself, Platt et al. provide evidence that activation does not necessarily interfere with receptor-mediated endocytosis [731]. Since the expression of adjuvant receptors is normally not restricted to one cell type, untargeted adjuvant administration can have considerable off-target effects including overstimulation of immune cells, uncontrolled cytokine release, pathological changes, such as splenomegaly and lymphadenopathy, or even a broad break of tolerance as well as organ destruction [732,733,734]. In particular, TLRs are also expressed on specific tumor types potentially rendering them resistant to apoptosis and enhance proliferation, invasion and metastasis following TLR signaling [735]. Parallel administration of antigen and adjuvant allows for the simultaneous entry of antigen and adjuvant into the DCs’ phagocytic system, which was demonstrated to be a pre-requisite for DCs to distinguish between pathogenic and self-antigens and to foster the formation of pMHC-II complexes [736].

Further, not all DCs may encounter antigen and adjuvant in parallel, which potentially leads to antigen presentation without co-stimulation. On the other hand, adjuvant stimulated DCs lacking foreign antigen could break tolerance against presented self-peptides [737,738,739]. Thus, techniques were developed to deliver antigen and adjuvant in parallel to DCs [740,741,742]. A study by Kreutz et al. revealed that cross-linking of αDEC205 targeting antibodies with the model antigen OVA and CpG-mimicking oligodeoxynucleotides (ODNs) was more efficient in priming cytotoxic CD8^+^ T cell responses than parallel administration of soluble ODNs and led to robust anti-tumor immune responses in the murine B16 melanoma model [742]. However, this study also demonstrated that following cross-linking, the attached ODNs as well as the antigen itself altered targeting specificity and binding properties of αDEC205-conjugates [742]. These issues can be overcome by simple spatial shielding of antigen and adjuvant within targeting antibody-labelled vehicles including polymer particles, liposomes or virus-like particles [695,701,705,743,744,745,746,747,748]. To further improve the efficacy of carrier systems to introduce antigens into the MHC-I presentation machinery, vehicles were developed displaying improved antigen escape into the cytosol or delivery into the endoplasmic reticulum (ER) [749,750,751]. While delivery of adjuvants to endosomal or plasma membrane located TLRs is simple, engineered cytosol or ER escape vehicle variants could be employed to efficiently deliver adjuvants to intracellular sensors including RLRs, NLRs, and STING or to enhance the cross-presentation ability [752]. Furthermore, such vehicles enable the targeted delivery of other regulatory molecules including small interfering (siRNAs), microRNAs, or RNAs coding for regulatory surface receptors or cytokines [753]. Targeted delivery of RNA coding for cytokines to DCs could be of particular interest, since in contrast to systemic application this would allow for the establishment of cytokine hotspots in close spatial proximity to DCs, rather than leading to systemic dilution following regular application. Further, delivery of RNA coding for the lymphoid homing receptor CCR7 might potentially restore the migration ability of cDCs, which in principle can be suppressed by the local tumor microenvironment [625]. In the case of moDCs, ex vivo introduction of RNA was already used for the generation of designer DCs optimized in cellular pathways, such as NF-κB signalling, suggesting that mRNA introduction into cDCs might bear allow for optimization of their functional properties [631,643,649]. With respect to silencing of regulatory genes via siRNA transduction, cellular RNA sensors might initiate off-target effects including type I IFN production. Owing to the observation, that delivery of short-hairpin RNAs only triggered RIG-I, but not TLR3, targeted delivery to cDC1 lacking RIG-I may constitute a promising approach for cDC1 manipulation without causing off-target effects [88,754]. A more detailed discussion of the different shuttle-systems is provided in other comprehensive reviews [755,756,757,758]. A selection of microRNAs (miRNAs) important for DC development and function was recently summarized elsewhere [1,759,760].

#### 6.2.3. Factors Contributing in Rationale for DC Vaccine Design

**Figure 4:**
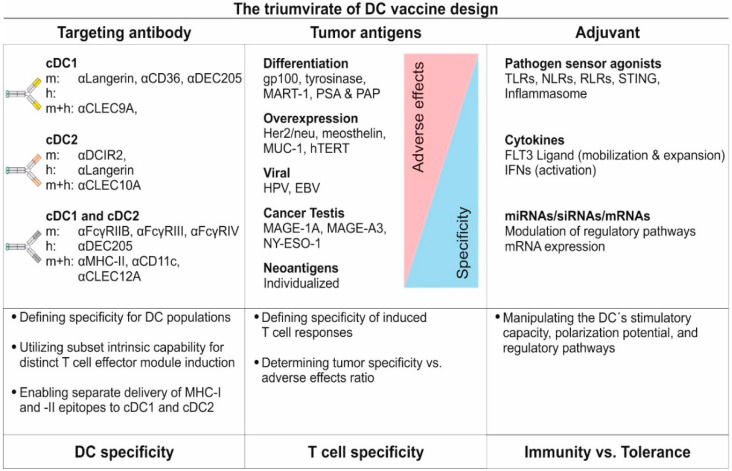
The rationale for targeted DC vaccine design is build up upon three pillars including the employed targeting antibody, the tumor antigen, and adjuvant(s). Antibody-based shuttle systems directed against specifically expressed endocytic receptors are a potent tool allowing for the delivery of antigens to specialized conventional dendritic cell (cDC) subpopulations. Thereby, the subset intrinsic capability to induce distinct T cell effector molecules would be utilized therapeutically. While targeting antibodies determine the specificity for a distinct cDC subpopulation, the selection of tumor antigens dictates the T cell specificity during vaccination approaches. Depending on the tumor, different antigen classes are available. Finally, the use of adjuvants determines the nature of the induced immune responses, thereby potentially allowing to break tolerance against tumor-derived antigens during peptide presentation on DCs to T cells. The manifold of adjuvant classes and effects enables complex manipulations of DCs. Adjuvants include pathogen sensor agonists stimulating and polarizing DC activity following sensing, direct application of cytokines mobilizing the DC pool or supporting DC activation, or delivery of miRNAs or siRNAs allowing for manipulation of regulatory pathways in DCs. Depending on the desired T cell immune response, the vaccine formulation should incorporate agents from every column. CLEC = C-type lectin receptor; Dendritic cell immuno-receptor 2 = DCIR2; FcγR = Fc gamma receptor; MHC = major histocompatibility complex; DC = d1endritic cell; cDC = conventional dendritic cell; gp100 = glycoprotein 100; Her2/neu = human epidermal growth factor 2; HPV = human papilloma virus; EBV = Epstein-Barr Virus; MAGE = Melanoma Antigen Gene; NY-ESO-1 = New York esophageal squamous cell carcinoma-1; TLR = Toll-like receptor; NLRs = Nucleotide-binding oligomerization domain-like receptors; RLR = RIG-I-like receptor; STING = Stimulator of interferon genes; FLT3 = Fms-like tyrosine kinase 3; FLT3-L = Fms-like tyrosine kinase ligand; IFN = Interferon; miRNA = micro RNA; siRNA = small interfering RNA.

The design of DC vaccines comprises three major building blocks as summarized in Figure 4. First, targeting antibodies can be utilized to address distinct DC subpopulations, thereby harnessing effector modules intrinsic to the individual DC subset. While the cross-presentation capability of murine cDC1 renders them ideal candidates for cytotoxic CD8+ T cell priming, parallel production of IL-12 and type I IFNs can foster Th1 cell, NK cell, NKT cell, and ILC1 induction boosting intracellular defense modules [543,577,761,762,763,764,765,766,767]. Thus, targeting murine cDC1 may be of particular importance. However, as we have already discussed, targeting of cDC2 was protective in a murine melanoma model rendering antigen delivery to cDC2 as an interesting approach to induce anti-tumor immunity [137]. This may be in particular true for the human system. There, the capability of cross-presentation and IL-12 production are differentially discussed in the literature [87,371,564,567,568,569,570,571,572].

Whereas targeting antibodies determine the DC specificity, antigen selection dictates T cell specificity for the tumor. Typical tumor antigens used for vaccination approaches stem from different classes. While the specificity from differentiation- and over-expressed antigens via viral and cancer-testis antigens to individual neo-antigens gradually increases, potential side effects and the danger of autoimmunity are minimized [106]. However, targeting neo-antigens requires individualized vaccines, thus not allowing for the production of classical off-the-shelf vaccines. Nevertheless, the use of neo-antigens might efficiently bypass thymic tolerance [768,769,770]. Furthermore, the initial identification of neo-antigens could become more feasible owing to newly developed sequencing techniques and elegant solutions for predicting the immunogenicity of different epitopes are already under development [771,772]. While single antigens can be either coupled to targeting antibodies or be included into nanovesicle systems, only vesicle systems enable the easy delivery of whole tumor cell lysates making the identification of cancer associated antigens dispensable [106]. However, delivery of tumor cell lysates exhibits major drawbacks including the necessity of initial isolation or availability of allogenic tumor material, many unknowns about the quality of included antigens or other compounds and potential delivery of non-tumor specific self-antigens. Since CD4+ T cell help is paramount for the induction and maintenance of productive cytotoxic CD8+ T cell responses, the inclusion of CD4 epitopes was pivotal in different studies utilizing peptide-based neo-antigen vaccines [771,773,774]. Furthermore, the presence of CD4+ T cell help in clinical cancer studies correlated with the induction of cytotoxic CD8+ T cell responses [771,773,774].

The last essential building block for DC vaccine formulation are adjuvants paramount for breaking tolerance against self-derived structures. For instance, select adjuvants further foster, reprogram and fine-tune both, T cell polarization and activation potential, of subset imprinted DC effector modules. Since the different cDC subpopulations display a discrete PRR expression pattern, inclusion of PAMP or DAMP mimicking adjuvants in antibody-coated vesicles either addressing cDC1 or cDC2, could also allow for subset-matched adjuvant delivery.

Finally, administration of factors mobilizing the cDC precursor pool might be beneficial, since application of FLT3-L expanded both, the pool of human pre-cDCs and fully developed cDCs in the circulation [685].

## 7. Combination Therapies—Potential and Hurdles

**Figure 5:**
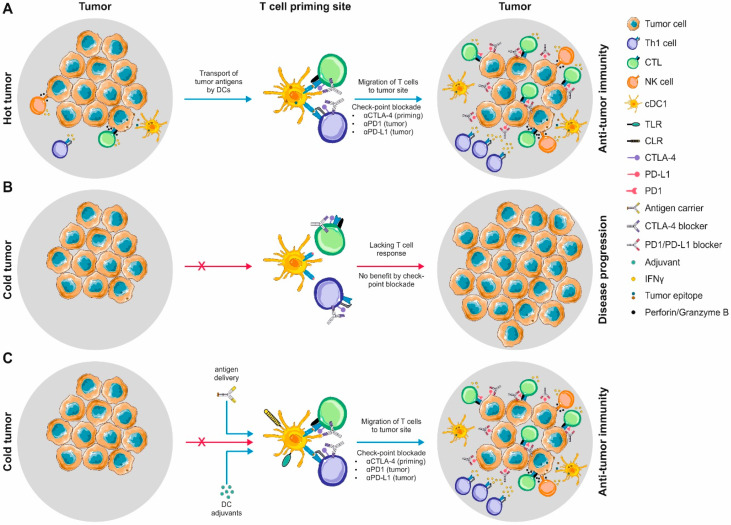
Combinatorial approaches manipulating antigen presentation of dendritic cells while removing the brakes from T cell immunity are a promising strategy in the treatment of malignant disease. (**A**) Malignancies can substantially differ in their overall signature including mutational load of tumor cells and the amount and quality of tumor infiltrating lymphocytes (TILs) together generating either an immunogenic or suppressive tumor microenvironment. Activity of cytotoxic CD8^+^ T cells (CTLs) and other supporting cell types, such as IFNγ producing CD4+ T helper type 1 cells (Th1) or natural killer (NK) cells, can lead to tumor cell lysis making tumor epitopes available for uptake by dendritic cells (DCs) in an inflammatory environment. Furthermore, NK cells are important for efficiently recruiting cDC1 to the tumor site. Following antigen cargo transport to lymphoid organs, DCs present tumor-derived epitopes to naïve T cells, thereby supporting the anti-tumor T cell effector pool. In the case of hot tumors, characterized by a high mutational burden and a quality immune infiltrate, minimal T cell responses can be amplified by therapeutic application of checkpoint inhibitors. Whereas αCTLA-4 treatment directly acts during priming of naïve T cells by DCs, blockade of PD-1 on T cells or PD-L1 on TILs or the tumor itself silences regulatory pathways in T cells in the tumor microenvironment. There, beside the tumor’s own capability to generate a suppressive microenvironment, mechanisms normally designed to keep inflammation in check including up-regulation of PD-1 or PD-L1 following recognition of IFNγ produced by CD4^+^ Th1 cells, cytotoxic CD8^+^ T cells and NK cells can be overruled. (**B**) Unfortunately, only a small pool of patients displays tremendous responses following checkpoint inhibitor monotherapy. This can potentially be explained by a low tumor mutational load, absence of TILs or presence of regulatory TILs, which are characteristics for cold tumors. Thus, tumor-derived antigens either become not available for DCs or are presented in an anti-inflammatory context leading to absence or regulatory T cell responses not allowing for amplification of T cell responses by checkpoint inhibition. (**C**) Hence, combinatorial approaches manipulating antigen accessibility and stimulatory capacity of DCs via antigen targeting and adjuvant application paralleled by silencing of regulatory T cell pathways may unleash the true power of immunotherapeutic approaches. Further, antigen targeting vanishes the need for initial tumor cell destruction or tumor antigen uptake and migration of DCs, since antigens can be delivered directly to lymphoid resident populations. There, parallel application of adjuvants leads to antigen presentation in an inflammatory context inducing productive T cell immunity. Finally, manipulation of DCs allowing for promotion of T cell memory responses may efficiently fight minimal residual disease owing to the generation of immunological memory. Note that CTLA-4 or PD-1 and PD-L1 only serve as prototypic examples for checkpoints mostly acting either during T cell priming or in the tumor microenvironment and only reflect a glance of the complexity of T cell therapy. CTLA-4 = Cytotoxic T lymphocyte antigen 4; PD-1 = programmed cell death protein 1; PD-L1 = programmed cell death protein 1 ligand 1. The cellular images are provided and adapted from Servier Medical Art (smart.servier.com). *The images are licensed under a Creative Commons Attribution 3.0 Unported License (creativecommons.org/licenses/by/3.0/)*.

Many proposed concepts for the application of DC vaccines in cancer therapies have demonstrated a limited efficacy in solid tumors. This means that the patients could benefit and might be even completely cured from the disease, but the proportion of these patients is comparably small, ranging from 7–32% [631,667]. In the meantime, the great success of checkpoint inhibitor therapy as monotherapy has shown that the immune system can efficiently fight human cancers. However, even the checkpoint inhibitor monotherapy is not working in all patients and its efficacy varies strongly (5–45%) between different tumor entities and stages [775,776]. Especially tumors demonstrating a high mutational load (leading to an easier detection by our immune system due to neo-epitope expression or stronger alteration in gene expression profiles) can be treated well by checkpoint-inhibitor monotherapy [777,778,779,780,781,782]. One explanation might be that the checkpoint blockade is acting mostly on pre-formed tumor-reactive T cells fostering their functionality [668,669,670]. However, some groups proposed a stronger induction of de novo T cell responses in a checkpoint-inhibitor regimen [669,783,784]. Of note, so-called immunological cold tumors, characterized by a low mutational burden and/or lacking or dysfunctional immune infiltrates, are hard to threat by any immunological therapy [615,625,780,785]. As a successful treatment of solid tumors needs both, T cells responding to tumor antigens as well as a high functionality and correct polarization of these T cell responses, combining DC vaccination strategies with checkpoint-inhibitor therapy is very attractive. However, the high number of different tumor entities, checkpoint-inhibitors, timing of the two therapies as well as potential vaccination strategies and targets leads to a high-dimensional room of possible combination therapies. Therefore, it is of utmost importance, to understand the underlying mechanisms, drawbacks, and potential of the antigen loading of DCs or other vaccination strategies as well as checkpoint-inhibition to find the missing links generating highly efficient immune responses, while minimizing potential side effects. A schematic model for the working principles of DC:T cell combination therapies is presented in Figure 5.

## 8. Summary of DC-Based Immunotherapeutic Approaches Unleashing Anti-Tumor Immunity

DC are the generals of adaptive immunity. They sense a wide variety of environmental cues including structures from pathogens and cancer cells. These are recognized by germline encoded, innate PRRs allowing for translation of the environment into distinct effector modules. In particular, induction of intracellular defense modules including NK cells, cytotoxic CD8^+^ T cells and Th1 cells, while avoiding Th2 and Th17 responses, can facilitate productive anti-tumor immunity. This is achieved by transport of tumor-derived cargo to secondary lymphoid organs stimulating T cell responses under inflammatory conditions. There, different approaches have been developed to regulate or fine-tune the induction of tumor immune responses at the DC level. To avoid antigen presentation without adequate co-stimulation, DCs can be manipulated with adjuvants to exert highly co-stimulatory functions during antigen presentation, thereby leading to productive immune responses. However, since the kind of environmental cue, in this case simulated by an adjuvant, is decrypted by DCs to polarize adequate T cells, adjuvants can be exploited to facilitate the induction of cytotoxic CD8^+^ T cells and Th1 cells, while hindering the induction of Th2 and Th17 responses, which are either unproductive or detrimental in the context of anti-tumor immunity. Further, T_reg_ cells are kept in check. In the case of tumors with reduced or absent immune infiltrates or impaired migration lacking the transport of tumor cargo to T cell priming sites, developed antigen delivery approaches may overcome this issue by directly loading DCs located in secondary lymphoid organs with tumor antigens. Although releasing the brakes of T cell immunity via checkpoint blockade emerged as prime treatment option of solid tumors in patients already exhibiting at least a minimal T cell response, a high percentage of patients do not benefit from checkpoint blockade. Therefore, prior initiation of antigen-specific T cell responses followed by checkpoint blockade represents a dynamic duo for cancer treatment. Additionally, other tissue-derived and cellular players have to be considered, which have been recently reviewed by the group of Merad [611].

However, despite the vast amount of great research conducted on targeted DC vaccination and checkpoint therapy, there is currently no consent on DC vaccine formulation and combination with specific checkpoint inhibitor treatment regimens. Thus, the era of combined DC and T cell-based immunotherapies has just begun.

## 9. Single Cell Omics Reshape Our Understanding of DC Biology Providing New Strategies for DC-Based Immunotherapeutic Approaches

Multiple studies have demonstrated that antigen targeting to DCs is a promising strategy for the treatment of malignant disease. New single-cell-based high resolution techniques, such as single-cell-RNA sequencing or mass cytometry, have led to the identification of phenotypic and functional heterogeneous subsets within the cDC2 population in mice and men exhibiting either pro- or anti-inflammatory properties [33,80,90,786]. Since these subsets exhibit a discrete expression of potential targeting receptors, including CLEC10A or FcγRIIB, this additional layer of complexity may enable the targeted manipulation of these pro- and anti-inflammatory cDC2 subsets [33,80]. Moreover, the increased resolution may be utilized to identify distinct cellular states beneficial or detrimental for disease outcome. Characterized states could serve as a blueprint for vaccine design rationale allowing for remodeling functional cell states by therapy on both, the DC and T cell site. Finally, parallel modulation of the immune system’s generals (DCs) and its soldiers (T cells) via therapeutic application of DC targeted vaccines and checkpoint manipulation may enable the treatment of diverse solid tumor entities with high response rates across patients.
